# Associations of long-term nitrogen dioxide exposure with a wide spectrum of diseases: a prospective cohort study of 0·5 million Chinese adults

**DOI:** 10.1016/S2468-2667(24)00264-0

**Published:** 2024-12-04

**Authors:** Xi Xia, Xia Meng, Cong Liu, Yi Guo, Xinyue Li, Yue Niu, Kin Bong Hubert Lam, Neil Wright, Christiana Kartsonaki, Yiping Chen, Ling Yang, Huaidong Du, Canqing Yu, Dianjianyi Sun, Jun Lv, Junshi Chen, Xiaoming Yang, Ruqin Gao, Shaowei Wu, Haidong Kan, Ka Hung Chan, Liming Li, Zhengming Chen, China Kadoorie Biobank Study Group, Junshi Chen, Zhengming Chen, Robert Clarke, Rory Collins, Liming Li, Jun Lv, Richard Peto, Robin Walters, Ahmed EdrisMohamed, Alfred Pozarickij, Andri Iona, Baihan Wang, Charlotte Clarke, Christiana Kartsonaki, Dan Schmidt, Daniel Avery, Derrick Bennett, Hannah Fry, Huaidong Du, Hubert Lam, Iain Turnbull, Iona Millwood, James Liu, Jonathan Clarke, Ka Hung Chan, Kshitij Kolhe, Kuang Lin, Lin Wang, Ling Yang, Maria Kakkoura, Maryam Rahmati, Maxim Barnard, Mohsen Mazidi, Neil Wright, Pang Yao, Paul Ryder, Pek Kei Im, Prapthi Harish, Qunhua Nie, Rebecca Stevens, Robert Clarke, Robin Walters, Ruth Boxall, Sam Morris, Simon Gilbert, Xiaoming Yang, Yiping Chen, Zhengming Chen, Xiao Han, Can Hou, Qingmei Xia, Chao Liu, Jun Lv, Pei Pei, Dianjianyi Sun, Canqing Yu, Lang Pan, Zengchang Pang, Ruiqin Gao, Shanpeng Li, Haiping Duan, Shaojie Wang, Yongmei Liu, Ranran Du, Yajing Zang, Liang Cheng, Xiaocao Tian, Hua Zhang, Yaoming Zhai, Feng Ning, Xiaohui Sun, Feifei Li, Silu Lv, Junzheng Wang, Wei Hou, Wei Sun, Shichun Yan, Xiaoming Cui, Chi Wang, Zhenyuan Wu, Yanjie Li, Quan Kang, Huiming Luo, Tingting Qu, Xiangyang Zheng, Zhendong Guo, Shukuan Wu, Yilei Li, Huimei Li, Ming Wu, Yonglin Zhou, Jinyi Zhou, Ran Tao, Jie Yang, Jian Su, Fang Liu, Jun Zhang, Yihe Hu, Yan Lu, Liangcai Ma, Aiyu Tang, Shuo Zhang, Jianrong Jin, Jingchao Liu, Mei Lin, Zhenzhen Lu, Lifang Zhou, Changping Xie, Jian Lan, Tingping Zhu, Yun Liu, Liuping Wei, Liyuan Zhou, Ningyu Chen, Yulu Qin, Sisi Wang, Xiangping Wu, Ningmei Zhang, Xiaofang Chen, Xiaoyu Chang, Mingqiang Yuan, Xia Wu, Xiaofang Chen, Wei Jiang, Jiaqiu Liu, Qiang Sun, Faqing Chen, Xiaolan Ren, Caixia Dong, Hui Zhang, Enke Mao, Xiaoping Wang, Tao Wang, Xi Zhang, Kai Kang, Shixian Feng, Huizi Tian, Lei Fan, Xiaolin Li, Huarong Sun, Pan He, Xukui Zhang, Min Yu, Ruying Hu, Hao Wang, Xiaoyi Zhang, Yuan Cao, Kaixu Xie, Lingli Chen, Dun Shen, Xiaojun Li, Donghui Jin, Li Yin, Huilin Liu, Zhongxi Fu, Xin Xu, Hao Zhang, Jianwei Chen, Yuan Peng, Libo Zhang, Chan Qu

**Affiliations:** a Department of Occupational and Environmental Health, School of Public Health, Xi'an Jiaotong University Health Science Center, Xi'an, China; b Key Laboratory of Environment and Genes Related to Diseases, Ministry of Education, Xi'an, China; c Clinical Trial Service Unit and Epidemiological Studies Unit, Nuffield Department of Population Health, University of Oxford, Oxford, UK; d School of Public Health, Shaanxi University of Chinese Medicine, Xi'an, China; e School of Public Health, Key Laboratory of Public Health Safety of the Ministry of Education and NHC Key Laboratory of Health Technology Assessment, Fudan University, Shanghai, China; f Department of Epidemiology and Biostatistics, School of Public Health, Peking University Health Science Center, Beijing, China; g Peking University Center for Public Health and Epidemic Preparedness & Response, Beijing, China; h Key Laboratory of Epidemiology of Major Diseases (Peking University), Ministry of Education, Beijing, China; i China National Center for Food Safety Risk Assessment, Beijing, China; j Qingdao Center for Disease Control and Prevention, Qingdao, China; k Children's Hospital of Fudan University, National Center for Children's Health, Shanghai, China

## Abstract

**Background:**

Little evidence is available on the long-term health effects of nitrogen dioxide (NO_2_) in low-income and middle-income populations. We investigated the associations of long-term NO_2_ exposure with the incidence of a wide spectrum of disease outcomes, based on data from the China Kadoorie Biobank.

**Methods:**

This prospective cohort study involved 512 724 Chinese adults aged 30–79 years recruited from ten areas of China during 2004–08. Time-varying Cox regression models yielded adjusted hazard ratios (HRs) for the associations of long-term NO_2_ exposure with aggregated disease incidence endpoints classified by 14 ICD-10 chapters, and incidences of 12 specific diseases selected from three key ICD-10 chapters (cardiovascular, respiratory, and musculoskeletal diseases) found to be robustly associated with NO_2_ in the analyses of aggregated endpoints. All models were stratified by age-at-risk (in 1-year scale), study area, and sex, and were adjusted for education, household income, smoking status, alcohol intake, cooking fuel type, heating fuel type, self-reported health status, BMI, physical activity level, temperature, and relative humidity.

**Findings:**

The analysis of 512 709 participants (mean baseline age 52·0 years [SD 10·7]; 59·0% female and 41·0% male) included approximately 6·5 million person-years of follow-up. Between 5285 and 144 852 incident events were recorded for each of the 14 aggregated endpoints. Each 10 μg/m^3^ higher annual average NO_2_ exposure was associated with higher risks of chapter-specific endpoints, especially cardiovascular (n=144 852; HR 1·04 [95% CI 1·02–1·05]), respiratory (n=73 232; 1·03 [1·01–1·05]), musculoskeletal (n=54 409; 1·11 [1·09–1·14]), and mental and behavioural (n=5361; 1·12 [1·05–1·21]) disorders. Further in-depth analyses on specific diseases found significant positive supra-linear associations with hypertensive disease (1·08 [1·05–1·11]), lower respiratory tract infection (1·03 [1·01–1·06]), arthrosis (1·15 [1·09–1·21]), intervertebral disc disorders (1·13 [1·09–1·17]), and spondylopathies (1·05 [1·01–1·10]), and linear associations with ischaemic heart disease (1·03 [1·00–1·05]), ischaemic stroke (1·08 [1·06–1·11]), and asthma (1·15 [1·04–1·27]), whereas intracerebral haemorrhage (1·00 [0·95–1·06]), other cerebrovascular disease (0·98 [0·96–1·01]), acute upper respiratory infection (1·03 [0·96–1·09]), and chronic lower respiratory disease (0·98 [0·95–1·02]) showed no significant association. NO_2_ exposure showed robust null association with external causes (n=32 907; 0·98 [0·95–1·02]) as a negative control.

**Interpretation:**

In China, long-term NO_2_ exposure was associated with a range of diseases, particularly cardiovascular, respiratory, and musculoskeletal diseases. These associations underscore the pressing need to implement the recently tightened WHO air quality guidelines.

**Funding:**

Wellcome Trust, UK Medical Research Council, Cancer Research UK, British Heart Foundation, National Natural Science Foundation of China, National Key Research and Development Program of China, Sino-British Fellowship Trust, and Kadoorie Charitable Foundation.

## Introduction

Nitrogen dioxide (NO_2_) is a reactive gaseous pollutant primarily produced during combustion processes and is often considered as a hallmark of transport-related air pollution along with ambient particulate matter with a diameter of <2·5 μm (PM_2·5_).^1^ Whereas long-term PM_2·5_ exposure was attributed as the top contributor to global disease burden in the Global Burden of Diseases, Injuries, and Risk Factors Study (GBD) 2021, NO_2_ was only considered as an attributable cause to asthma in the same study.^2^

Growing evidence suggests adverse health impacts from long-term exposure to NO_2_,^1^, ^3^, ^4^, ^5^, ^6^ but important knowledge gaps remain. A 2024 systematic review of 56 cohort studies found 3–7% higher risks of all-cause, cardiovascular, and respiratory mortality per 10 μg/m^3^ higher annual NO_2_ exposure.^3^ Long-term NO_2_ exposure has also been consistently associated with asthma incidence, but prospective evidence on other diseases (eg, cardiovascular disease and depression) is scarce.^1^, ^4^, ^5^ Of note, most modern cohort studies were conducted in high-income countries (HICs) with low NO_2_ exposure, examined few health outcomes, or involved small samples.^1^, ^4^, ^5^, ^6^

**Research in context**

**Evidence before this study**

We searched PubMed for articles published in English from Jan 1, 2000, to Aug 24, 2024, using the terms (“nitrogen dioxide” or “NO2”) AND “cohort” AND (“incidence” or “disease” or “hospitali*tion”). We found studies on overall and cardio-respiratory mortality, and on multiple individual diseases in the same body system, but no prospective cohort studies simultaneously examining the associations of long-term nitrogen dioxide (NO_2_) exposure with incidences of diseases across multiple systems. Most previous studies were conducted in high-income populations, with the exception of a few cohort studies (n<50 000) examining cardiovascular and neurodegenerative diseases in China. A 2024 systematic review reported that long-term NO_2_ exposure was associated with a 3–7% higher risk of all-cause, cardiovascular, and respiratory mortality, whereas a 2022 systematic review by the Health Effect Institute found inconclusive evidence on the associations of long-term NO_2_ exposure with disease incidence (except for asthma) from cohort studies. Consistently, the Global Burden of Diseases, Injuries, and Risk Factors Study 2021 found sufficient evidence only to attribute asthma incidence but no other diseases to NO_2_ exposure.

**Added value of this study**

To our knowledge, this is the first large, prospective study to systematically and simultaneously investigate the associations of NO_2_ with a wide range of conditions in any low-income and middle-income countries with high NO_2_ exposure. By combining high-resolution spatiotemporal models of ambient NO_2_ exposure with extensive epidemiological data (6·5 million person-years of follow-up) in 0·5 million adults in the China Kadoorie Biobank, we found long-term NO_2_ exposure to be associated with higher risks of incidences of cardiovascular disease, respiratory disease, musculoskeletal disease, and mental and behavioural disorders after extensive adjustment for key confounders in single-pollutant models. The associations with cardiovascular, respiratory, and musculoskeletal diseases persisted upon additional adjustment for particulate matter with a diameter of less than 2·5 μm (PM_2·5_) or ozone exposure, across different cumulative lags (0–3 and 0–5 years), and in individuals without previous history of major diseases. Subgroup analyses suggested that people older than 60 years and female individuals might be particularly susceptible to the adverse impact of NO_2_.

**Implications of all the available evidence**

Growing evidence suggests that long-term NO_2_ exposure has adverse effects on multiple body systems and thus increases risks of developing a wide range of diseases, probably independently of PM_2·5_. The disease burden attributed to NO_2_ could be largely underestimated. The current findings lend support to the recently tightened WHO air quality guidelines and, in particular, the associated effort in controlling traffic-related air pollution, which is a major source of NO_2_ in most populations. However, further research is needed to investigate the impact of NO_2_ on a wider range of specific diseases not covered in the current literature, NO_2_ exposure from indoor sources, and the complex interaction between NO_2_ and other co-pollutants.

In 2021, WHO tightened the air quality guideline for annual average NO_2_ from 40 μg/m^3^ to 10 μg/m^3^.^7^ In recent decades, NO_2_ exposure has been declining rapidly in HICs, but increasing in many low-income and middle-income countries (LMICs) alongside considerable car fleet expansion.^1^ However, relevant cohort studies in LMICs remain scarce, and the few existing studies had small sample sizes (n<50 000) and focused on a few mortality or disease outcomes.^4^, ^5^, ^8^, ^9^ To explore the health impact of NO_2_ in LMICs, we conducted one of the largest systematic aetiological investigations of the associations of long-term NO_2_ exposure with incidence of a wide spectrum of disease outcomes, based on data from the China Kadoorie Biobank (CKB).^10^

## Methods

### Study design and participants

Details of CKB have been described elsewhere.^10^, ^11^ Briefly, this prospective cohort study recruited 512 724 adults aged 30–79 years via multistage cluster sampling during 2004–08, from ten diverse areas across China, with 100–150 administrative units (rural villages or urban street committees) in each study area (appendix p 19). In each administrative unit, a survey clinic was set up at a central location within approximately 1 km from the residences of most eligible participants. At recruitment, trained interviewers administered a laptop-based questionnaire covering information on sociodemographics (eg, sex as indicated on national ID card), lifestyle (eg, smoking and physical activity), environmental exposure (eg, solid fuel use), and medical characteristics, and took physical measurements (eg, height, weight, and blood pressure) following standardised protocols.

The study was approved by the Ethical Review Committee of the Chinese Center for Disease Control and Prevention (Beijing, China; 2018-1038) and the Oxford Tropical Research Ethics Committee, University of Oxford (Oxford, UK; 5109-17). All participants provided written informed consent upon recruitment.

### Assessment of air pollution exposure

As described previously,^12^ we estimated ground-level NO_2_ concentrations across mainland China with a high-resolution (1 km × 1 km) satellite-based random-forest model that integrated the POMINO-TROPOMI NO_2_ vertical column density data derived from the TROPOspheric Monitoring Instrument onboard the Sentinel-5 Precursor satellite, and simulated NO_2_ concentrations based on the community multiscale air quality model,^13^ meteorological parameters (eg, air temperature and relative humidity), and other ancillary variables (eg, population density and road networks). The model effectively captured spatial variability and was validated against ground-level NO_2_ measurements, with an overall cross-validation *R*^2^ of 0·72 (root mean square error 10·5 μg/m^3^) during 2013–18.^12^ For the present study, annual average NO_2_ concentrations by 1 km × 1 km grid were assigned to participants per year of follow-up, from 2005 to the year of study outcome incident or censoring, according to the participants’ baseline study clinic location. We also applied well validated satellite-based random-forest models in estimating PM_2·5_ (cross-validation *R*^2^=0·81)^11^, ^14^ and ozone (O_3_; 0·83)^15^ following similar approaches, for adjustment in two-pollutant models. Details of these models, including the range of sources of air pollution considered, have been described previously^11^, ^14^, ^15^ and are summarised in the appendix (pp 4–6).

### Follow-up and incidence outcomes

After the baseline survey, participants were continuously followed up for death and hospitalisation via electronic linkages to death and disease registries and national health insurance databases (with >96% coverage of relevant events).^16^ All events were coded according to ICD-10 by trained staff who were masked to participants’ baseline information. Participants without the outcome of interest were censored upon death, at loss to follow-up, or on Dec 31, 2018, whichever came first. By Dec 31, 2018, 56 536 participants had died (including 13 during 2004) and 4028 (<1%) were lost to follow-up. Further details on outcome ascertainment, disease adjudication, and rationale to censor at death (for competing risk) are included in the appendix (p 7).

Disease incidences (ie, first events of hospitalisation or death during the follow-up) were first classified according to 13 individual ICD-10 chapter endpoints (see appendix p 8 for the rationale of examining composite incidence), along with an aggregated incidence endpoint of external causes (combining two ICD-10 chapters: XIX [injury, poisoning and certain other consequences of external causes] and XX [external causes of morbidity and mortality]) included as a negative control to detect residual confounding from neighbourhood-level factors (eg, residential proximity to main roads), totalling 14 aggregated endpoints (appendix p 13). At baseline, CKB ascertained self-reported medical history of diseases involving only eight ICD-10 chapters, so the main analyses avoided differential exclusions of prevalent cases, but were supplemented by sensitivity analyses applying exclusions where possible.

### Statistical analysis

We excluded participants who died before 2005 (n=13; due to the lack of reliable exposure data before 2005) or who had missing data for any covariates (n=2 with missing BMI), leaving 512 709 participants. We further restricted the prospective analyses to age-at-risk of 35–85 years by disease endpoint, to capture the likely preventable impact of NO_2_ exposure in middle-aged to older-aged adults (appendix p 20).

Age-adjusted, sex-adjusted, and study area-adjusted percentages or means of baseline characteristics were calculated by quartiles of NO_2_ exposure at baseline. Cox proportional hazard models were used to estimate hazard ratios (HRs) for disease incidence associated with long-term NO_2_ exposure, with annual average concentrations included as a time-varying variable (ie, one value per follow-up year). NO_2_ was fitted as a linear term (per 10 μg/m^3^ increase across the 1st and 99th percentile exposure range, to reduce the effect of unstable estimates from outliers) to enable comparison with previous studies, and as a penalised spline function with three degrees of freedom in separated models to graphically assess potentially non-linear associations. All models were stratified by age-at-risk (in 1-year scale), ten study areas (to account for differences in exposure, covariates, and disease patterns), and sex (if applicable), and were adjusted for education (no formal school, primary school, middle school, or high school or above), household income (<20 000 ¥/year, 20 000–34 999 ¥/year, or ≥35 000 ¥/year), smoking status (never regular, occasional, ex-regular, or current regular), alcohol intake (never regular, ex-regular, occasional or seasonal, monthly, reduced intake [defined as previously weekly but now occasionally], or weekly), cooking and heating fuel exposure (always clean, solid to clean, always solid, never cooking or never heating, and others; solid fuels included wood, charcoal, and coal, and clean fuels included gas, electricity, and district heating [for heating only]), self-reported health status (excellent, good, fair, or poor), BMI (continuous), physical activity level (in metabolic equivalent of task-hours per day [MET-h/day]), and annual average temperature (continuous) and relative humidity (continuous; appendix pp 9–10). No violation of the proportional hazard assumption was observed from Schoenfeld residuals tests.

For each ICD-10 chapter, subgroup analyses were performed by age, sex, smoking status, alcohol intake, and median MET-h/day. Additionally, we fitted two-pollutant models by introducing ambient PM_2·5_ (main + PM_2·5_ model) or O_3_ (main + O_3_ model) into the main single-pollutant models to explore potential confounding from co-pollutants (appendix p 11). For further investigation, 12 specific diseases with more than 3000 incident events each were selected from three ICD-10 chapters that showed consistently significant associations with NO_2_ in the main and sensitivity analyses (appendix pp 12, 14). To assess the potential impact (eg, reverse causation) of prevalent or subclinical conditions at baseline, we also conducted sensitivity analyses by excluding participants with incidence recorded during the first 3 follow-up years or those with medical history of relevant conditions at baseline separately. To explore the possibility of time-lagged associations, we examined NO_2_ exposure at lag 0–3 years and lag 0–5 years. Cochran's Q test was used to compare associations by subgroup and between the main and sensitivity analyses.

Based on GBD 2019,^17^ we calculated the population attributable fraction (PAF) for incidences of three ICD-10 chapters showing significant associations with NO_2_ exposure consistently across the main and sensitivity analyses. The highest WHO air quality guideline target of annual average NO_2_ concentration (ie, <10 μg/m^3^) was used as our reference level. The PAF was calculated as:

$$PAF=\frac{\int_{x=ref}^{max}HR\left(x\right)P\left(x\right)-1}{\int_{x=ref}^{max}HR\left(x\right)P\left(x\right)}$$

where *HR(x)* is the exposure–response function from the main Cox models, with the reference level as *ref* and the highest level as *max*, and *P(x)* is the distribution function of NO_2_ exposure in CKB. All analyses were conducted in R (version 4.3.2).

### Role of the funding source

The funders of the study had no role in study design, data collection, data analysis, data interpretation, or writing of the report.

## Results

**Figure 1:**
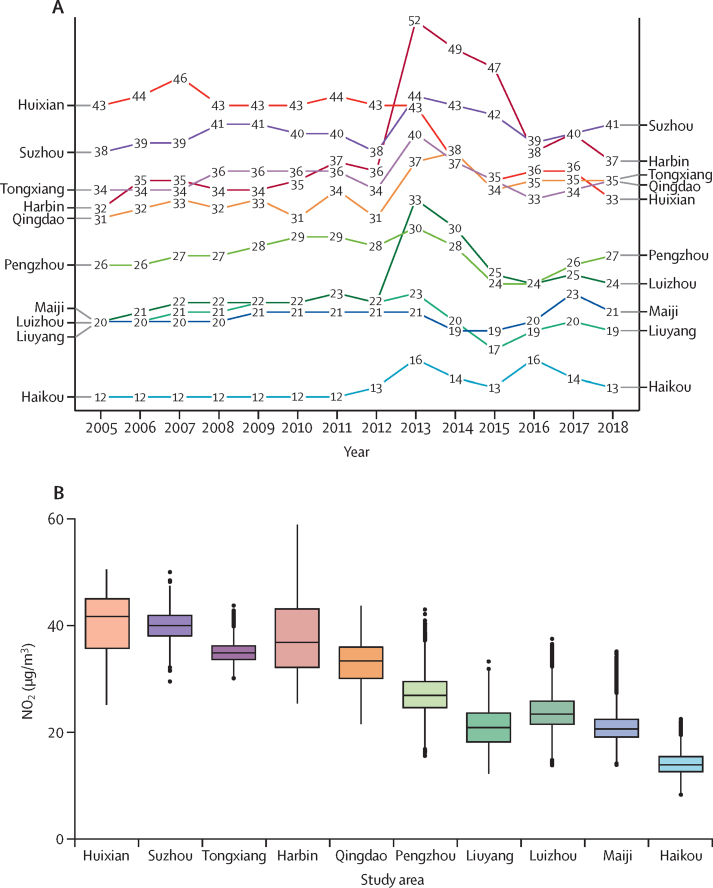
Annual average ambient NO_2_ concentration (A) and area-specific distribution of NO_2_ concentrations (B) from 2005 to 2018 across ten study areas In panel B, study areas are ranked in descending order of annual average NO_2_ levels in 2005. In the box plots, horizontal bolded lines represent medians across the whole 2005–18 period and the edges of the boxes represent IQRs. Vertical lines indicate 1·5 times the IQR. Black dots indicate outliers. NO_2_=nitrogen dioxide.

Of the 512 709 participants analysed (mean baseline age 52·0 years [SD 10·7]; 59·0% female and 41·0% male; 44·1% urban residents), in 2005–08 the annual average NO_2_ exposure ranged from 19·0 μg/m^3^ (SD 3·0) in the first quartile to 41·1 μg/m^3^ (2·9) in the fourth quartile, with 99·96% of person-years recording exposure above 10 μg/m^3^. Participants with higher NO_2_ exposure tended to reside in urban areas, have higher household income, consume alcohol weekly, use clean fuels for cooking or heating, and have higher exposure to PM_2·5_ (table). NO_2_ was strongly positively correlated with PM_2·5_ and weak-to-moderately inversely correlated with O_3_ across study areas (with a few exceptions—eg, in Harbin and Qingdao, where strong inverse correlation was observed; appendix p 15). Overall mean NO_2_ concentrations during 2005–18 varied considerably across and, to a lesser extent, within study areas, ranging from 12·9 μg/m^3^ (2·2) in Haikou to 39·9 μg/m^3^ (2·9) in Suzhou (figure 1). Generally, the NO_2_ levels for most study areas increased considerably during 2005–13, followed by a slight decline, resulting in a modest rise across the whole follow-up period (figure 1A).

**Table:** Baseline characteristics of participants by quartiles of ambient NO_2_ exposure averaged from 2005 to 2008

		**NO**_2_**concentration, μg/m^3^**				**Total (n=512 709)**
		≤21·4 (n=131 343)	>21·4 to ≤29·8 (n=139 963)	>29·8 to ≤37·3 (n=118 094)	>37·3 (n=123 309)	
Age, years		51·7 (11·0)	51·7 (10·8)	51·6 (10·3)	52·1 (10·5)	52·0 (10·7)
Sex						
	Female	59·2%	57·1%	57·8%	60·8%	59·0%
	Male	40·8%	42·9%	42·2%	39·2%	41·0%
Urban area		32·6%	46·4%	39·2%	55·1%	44·1%
Household income, ¥/year						
	<20 000	71·8%	63·2%	61·4%	60·9%	57·3%
	20 000–34 999	17·6%	24·4%	22·1%	21·3%	24·7%
	≥35 000	10·6%	12·4%	16·5%	17·8%	18·0%
Education						
	No formal school	18·3%	12·1%	23·6%	14·6%	18·6%
	Primary school	39·1%	31·6%	30·8%	27·1%	32·2%
	Middle school	25·1%	31·0%	25·6%	33·2%	28·3%
	High school or above	17·5%	25·3%	20·0%	25·1%	20·9%
Smoking status						
	Never regular	62·8%	60·9%	61·2%	60·0%	61·9%
	Occasional	5·6%	5·8%	6·0%	7·4%	5·7%
	Ex-regular	4·7%	6·2%	6·4%	6·7%	6·0%
	Current regular	26·9%	27·2%	26·4%	25·9%	26·4%
Alcohol intake						
	Never regular	52·4%	42·8%	41·8%	34·1%	45·9%
	Ex-regular	2·2%	2·1%	1·6%	1·9%	1·8%
	Occasional or seasonal	28·8%	33·4%	36·0%	37·1%	31·8%
	Monthly	2·3%	3·5%	3·6%	4·0%	3·4%
	Reduced intake	2·4%	2·6%	2·1%	2·5%	2·3%
	Weekly	11·9%	15·7%	14·8%	20·3%	14·8%
Heating fuels						
	Always clean	5·9%	10·2%	10·4%	11·8%	8·6%
	Solid to clean	2·9%	19·2%	13·7%	20·9%	11·3%
	Always solid	51·1%	42·2%	31·8%	25·4%	36·2%
	No heating	35·3%	22·8%	38·5%	35·2%	38·9%
	Others	4·8%	5·6%	5·6%	6·7%	5·0%
Cooking fuels						
	Always clean	11·3%	19·5%	16·3%	19·1%	17·0%
	Solid to clean	15·5%	20·2%	17·9%	23·1%	20·5%
	Always solid	47·4%	35·4%	38·2%	32·1%	36·0%
	No cooking	22·5%	20·8%	23·3%	20·3%	22·2%
	Others	3·3%	4·1%	4·3%	5·4%	4·3%
Physical activity, MET-h/day		21·1 (12·8)	20·0 (12·0)	22·7 (15·2)	19·4 (15·1)	21·1 (13·9)
BMI, kg/m^2^		23 (3·2)	23·7 (3·3)	23·7 (3·5)	24·3 (3·4)	23·7 (3·4)
Self-reported health status						
	Excellent	12·2%	18·4%	18·7%	22·6%	17·6%
	Good	24·5%	24·8%	30·3%	25·7%	28·1%
	Fair	50·8%	45·7%	39·2%	39·9%	43·9%
	Poor	12·5%	11·1%	11·8%	11·8%	10·4%
PM_2·5_, μg/m^3^		55·2 (8·0)	61·6 (3·8)	64·1 (3·8)	71·4 (9·2)	62·9 (10·2)
O_3_, μg/m^3^		75·4 (2·5)	73·5 (6·5)	75·2 (6·3)	74·8 (8·9)	76·0 (6·8)
NO_2_, μg/m^3^		19·0 (3·0)	25·5 (2·6)	34·8 (1·6)	41·1 (2·9)	29·8 (9·5)
Temperature, °C		17·3 (5·4)	14·5 (5·6)	13·5 (4·4)	13·7 (3·9)	15·6 (5·1)
Relative humidity, %		72·0 (6·0)	67·4 (5·4)	65·4 (3·8)	63·6 (6·5)	67·4 (6·6)

Categorical variables are presented as percentages and continuous variables are presented as mean (SD). All means and percentages in the four NO_2_ groups were adjusted for age, sex, and study area, where appropriate. The cutoffs of quartiles of NO_2_ levels are rounded to the nearest one decimal place. NO_2_=nitrogen dioxide. MET-h/day=metabolic equivalent of task-hours per day. PM_2·5_=particulate matter with a diameter of <2·5 μm. O_3_=ozone.

**Figure 2:**
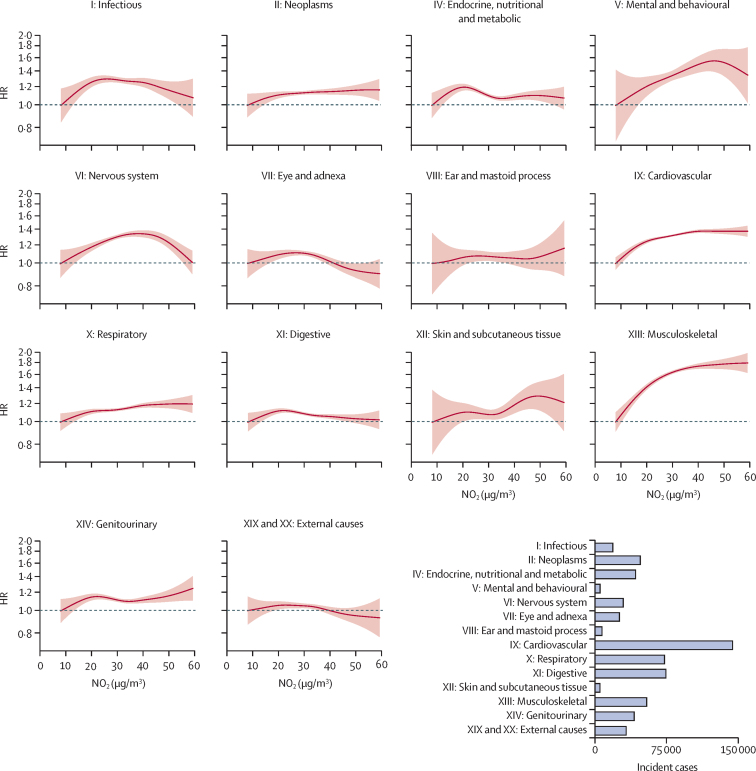
Exposure–response relationships of long-term NO_2_ exposure with disease incidence across 14 ICD-10 chapter-based endpoints in the main models Solid lines represent HRs and the shaded areas represent 95% CIs. All models were stratified by age-at-risk (in 1-year scale), ten study areas, and sex, and were adjusted for education, household income, smoking status, alcohol intake, cooking fuel type, heating fuel type, self-reported health status, BMI, physical activity level, temperature, and relative humidity. The bottom right panel shows the number of incident cases recorded for each aggregated endpoint during the follow-up period. HR=hazard ratio. NO_2_=nitrogen dioxide.

**Figure 3:**
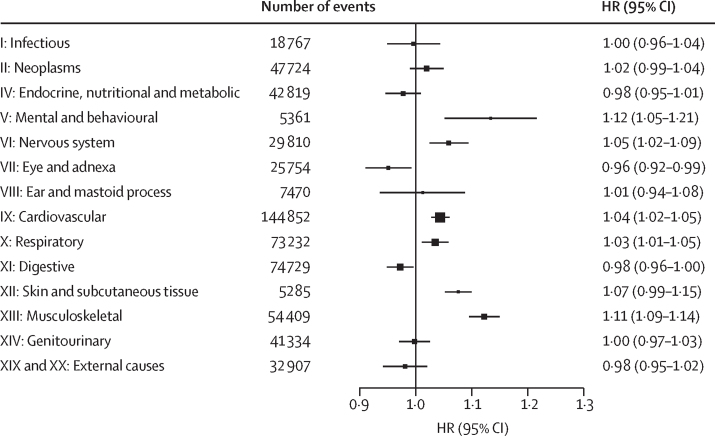
Hazard of 14 ICD-10 chapter-based endpoints per 10 μg/m^3^ higher annual NO_2_ exposure in the main models The solid boxes represent HRs, with the size inversely proportional to the variance of the logarithm of the HR, and the horizontal lines represent 95% CIs. All models were stratified by age-at-risk (in 1-year scale), ten study areas, and sex, and were adjusted for education, household income, smoking status, alcohol intake, cooking fuel type, heating fuel type, self-reported health status, BMI, physical activity level, temperature, and relative humidity. The models fit from the 1st to 99th percentiles of NO_2_ concentration. HR=hazard ratio. NO_2_=nitrogen dioxide.

During approximately 6·5 million person-years of follow-up, the top causes of incident events were cardiovascular (n=144 852), digestive (n=74 729), respiratory (n=73 232), and musculoskeletal (n=54 409) diseases (appendix p 13). In the main models, annual NO_2_ exposure showed linear or supra-linear associations with mental and behavioural (HR 1·12 [95% CI 1·05–1·21]), cardiovascular (1·04 [1·02–1·05]), respiratory (1·03 [1·01–1·05]), and musculoskeletal (1·11 [1·09–1·14]) diseases, but no clear association with other disease categories (Figure 2, Figure 3). Additional adjustment for PM_2·5_ resulted in generally stronger positive associations, and the associations with cardiovascular, respiratory, and musculoskeletal diseases persisted, with suggestive evidence of additional significant associations with infectious (per 10 μg/m^3^ NO_2_: HR 1·08 [95% CI 1·03–1·13]), ear and mastoid process (1·23 [1·14–1·33]), digestive (1·04 [1·02–1·07]), and genitourinary (1·07 [1·04–1·11]) diseases (appendix pp 16, 21). By contrast, associations did not change materially in the main + O_3_ models (Cochrane's Q test p>0·05 for all; appendix pp 16, 21). Across all three models, NO_2_ had no association with external causes (Figure 2, Figure 3; appendix p 21).

**Figure 4:**
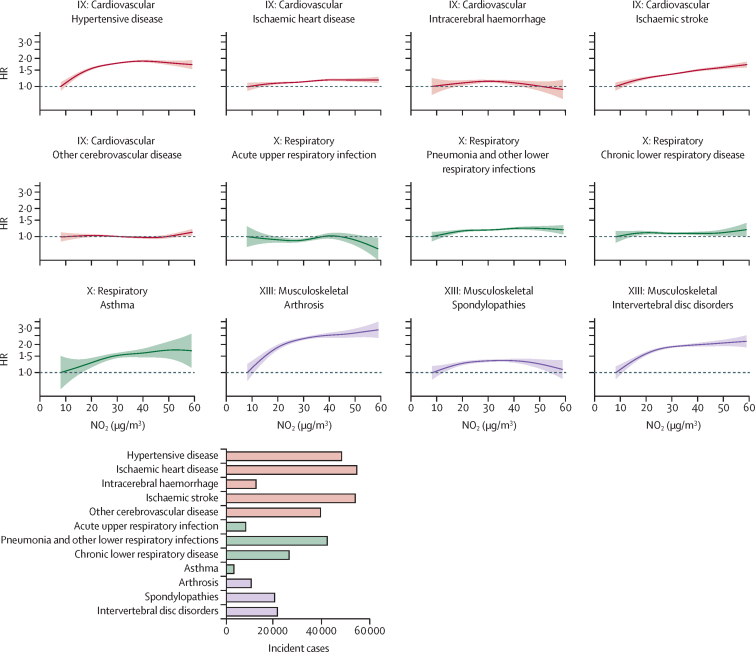
Exposure-response relationships of long-term NO_2_ exposure with 12 specific causes of disease incidence in the main models Solid lines represent HRs and shaded areas represent 95% CIs. All models were stratified by age-at-risk (in 1-year scale), ten study areas, and sex, and were adjusted for education, household income, smoking status, alcohol intake, cooking fuel type, heating fuel type, self-reported health status, BMI, physical activity level, temperature, and relative humidity. The bottom panel shows the number of incident cases of the 12 specific endpoints across three ICD-10 chapters. HR=hazard ratio. NO_2_=nitrogen dioxide.

**Figure 5:**
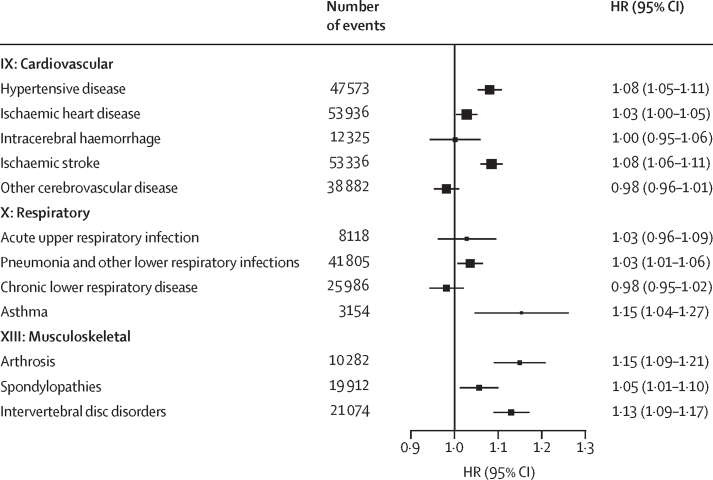
Hazard of 12 specific causes per 10 μg/m^3^ higher annual NO_2_ exposure in the main models The solid boxes represent HRs, with the size inversely proportional to the variance of the logarithm of the HR, and the horizontal lines represent 95% CIs. All models were stratified by age-at-risk (in 1-year scale), ten study areas, and sex, and were adjusted for education, household income, smoking status, alcohol intake, cooking fuel type, heating fuel type, self-reported health status, BMI, physical activity level, temperature, and relative humidity. The models fit from the 1st to 99th percentiles of NO_2_ concentration. HR=hazard ratio. NO_2_=nitrogen dioxide.

Of the 12 specific diseases across three selected ICD-10 chapters, hypertensive disease, lower respiratory infection, arthrosis, spondylopathies, and intervertebral disc disorders showed supra-linear positive associations, and ischaemic heart disease, ischaemic stroke, and asthma showed linear positive associations, whereas intracerebral haemorrhage, other cerebrovascular disease, acute upper respiratory infection, and chronic lower respiratory disease showed null associations with annual NO_2_ exposure in the main models (Figure 4, Figure 5). These associations again largely persisted in the two-pollutant models (appendix p 22).

The exposure–response associations of NO_2_ with most disease endpoints in the main models were consistent across subgroups examined, with a few notable exceptions (appendix pp 23–32). Specifically, the association with mental and behavioural disorders was stronger in male participants than female participants (p_heterogeneity_<0·001; appendix pp 25–26) and in ever-regular smokers than never-regular smokers (p_heterogeneity_<0·001; appendix pp 27–28). The association with musculoskeletal disease was significantly stronger in female participants than male participants (p_heterogeneity_<0·001; appendix pp 25–26), in never-regular smokers than ever-regular smokers (p_heterogeneity_<0·001; appendix pp 27–28), and in participants with never-regular alcohol intake than in those with ever-regular alcohol intake (p_heterogeneity_<0·001; appendix pp 31–32). However, the subgroup differences by smoking or drinking status diminished when the analysis was restricted to male participants (appendix p 33).

Compared with the main models, sensitivity analyses excluding individuals with relevant disease incidence during the first 3 follow-up years support the robustness of most associations, with the exception of that with mental and behavioural disorders (HR 1·06 [95% CI 0·98–1·14] *vs* 1·12 [1·05–1·21] in the main model; appendix p 34). Additionally, excluding individuals with previous medical history at baseline (for eight chapters) did not alter the results materially, with the exception of endocrine, nutritional and metabolic disease (appendix pp 17, 35). When examining NO_2_ exposure by lag 0–3 years and lag 0–5 years, the associations with cardiovascular, respiratory, musculoskeletal, nervous system, and eye and adnexa diseases persisted, but the association with mental and behavioural diseases attenuated to the null, and a few outcomes with null associations in the main analysis showed marginal positive associations (appendix p 36).

If the observed associations that were consistent across the main and sensitivity analyses were assumed causal, the PAFs attributed to annual average NO_2_ exposure at ≥10 μg/m^3^ were 18·3% (95% CI 16·9–19·7) for cardiovascular disease, 6·8% (4·5–9·0) for respiratory disease, and 32·0% (30·1–33·9) for musculoskeletal disease (appendix p 18).

## Discussion

In this large prospective cohort of 0·5 million Chinese adults, long-term NO_2_ exposure showed consistently significant positive associations with disease incidence across three ICD-10 chapters (ie, cardiovascular, respiratory, and musculoskeletal diseases) and robust null association with external causes across multiple models and sensitivity analyses. In disease-specific analyses, NO_2_ was associated with increased risks of eight (hypertensive disease, ischaemic heart disease, ischaemic stroke, lower respiratory infection, asthma, arthrosis, spondylopathies, and intervertebral disc disorders) of the 12 common diseases studied. Most associations observed were linear or supra-linear, with no apparent lower threshold of adversity down to the latest WHO air quality guideline level (10 μg/m^3^), highlighting the importance of reducing NO_2_ pollution.

Most previous cohort studies on NO_2_ have focused on all-cause, cardiovascular, or respiratory mortality, with the latest systematic review of 56 cohorts reporting significant (3–7%) elevated risks per 10 μg/m^3^ higher annual average exposure.^3^ However, studies of disease incidence remain crucial to understand the role of NO_2_ in disease development, with less unaccounted confounding (eg, quality of treatment) or reverse causality (eg, post-disease incidence behavioural and exposure changes).^18^ In particular, long-term NO_2_ exposure in adults has been largely consistently associated with asthma incidence, but the associations with other diseases have been inconclusive,^1^, ^4^, ^5^, ^6^ and cohort studies in LMICs are scarce, with most assessing a few disease outcomes at a time.^4^, ^5^, ^8^, ^9^ To the best of our knowledge, this is the first large-scale, systematic investigation of the associations of long-term NO_2_ exposure with a wide spectrum of disease incidence outcomes in LMICs. We found long-term NO_2_ exposure to be associated with modestly (3–5%) higher risks of incidence from cardiovascular and respiratory diseases, which is coherent with previous mortality studies.^3^

For cardiovascular disease-related outcomes, the only previous Chinese cohort (n=36 948) other than CKB reported more extreme HRs of 1·56 (95% CI 1·48–1·64) for total cardiovascular disease (4428 cases), 1·52 (1·42–1·63) for hypertension (2448 cases), and 1·66 (1·49–1·87) for stroke (1044 cases) per 10 μg/m^3^ higher annual ambient NO_2_ exposure.^5^ These findings are in stark contrast to cohort evidence from HICs, which showed null associations for ischaemic heart disease and total stroke in a 2022 systematic review.^1^ Apart from the risk of small sample bias, which tends to result in inflated associations, the Chinese study used a relatively crude (10 km × 10 km) NO_2_ prediction model matched to 162 county-level localities across China and defined disease incidence via self-reported doctor diagnosis.^5^ By contrast, with almost 145 000 well characterised cardiovascular disease incident cases (eg, 93% of stroke cases were confirmed by brain imaging^19^), we employed a well validated, 1 km × 1 km model matched to participants’ residential neighbourhood, and adjusted for a wide range of confounders. The association between NO_2_ and cardiovascular disease showed little subgroup difference, except a modestly stronger association in ever-regular drinkers, but cautious interpretation is required.

In disease-specific analyses, NO_2_ was associated with hypertensive disease, ischaemic heart disease, and ischaemic stroke, but not intracerebral haemorrhage, suggesting a common aetiological relevance to ischaemic but not haemorrhagic diseases, which is broadly consistent with our previous findings on PM_2·5_.^11^ These contrasting associations were not investigated in the previous Chinese cohort,^5^ whereas few previous studies in HICs have examined ischaemic stroke and intracerebral haemorrhage separately,^20^ with a recent Danish cohort reporting null associations.^21^ However, studies in HICs generally examined a narrow exposure spectrum and recorded few events. Our novel observations should be further verified in large-scale cohort studies in China or other LMICs.

For respiratory disease, NO_2_ has been associated with a 10–17% higher risk (per 10 μg/m^3^) of adult asthma incidence in a 2022 Health Effects Institute meta-analysis^1^ and a 2021 cohort study (ELAPSE), with evidence of levelling off at more than 30 μg/m^3^ NO_2_.^18^ In CKB, NO_2_ was associated with a 15% (95% CI 4–27) higher risk of asthma incidence, with a flattening association at greater than 35 μg/m^3^, lending further support to previous studies. Existing evidence on chronic obstructive pulmonary disease (COPD), however, has been inconsistent, with the Health Effect Institute meta-analysis reporting a null association, but two Scandinavian cohorts showing modest positive associations (10–13% higher risk).^6^, ^22^ Our findings support the Health Effect Institute review, with no significant association observed for chronic lower respiratory disease, a composite endpoint probably capturing most COPD incidents requiring hospitalisation in CKB.^23^ The discrepancies could be due to differences in outcome definition, with the Scandinavian administrative cohorts^6^, ^22^ capturing not only inpatient (as in most other cohorts including CKB) but also milder outpatient cases, whereas COPD is under-diagnosed in China.^23^ Cohort evidence on other respiratory diseases has been scarce, with two Danish cohorts reporting significant positive associations (7–17% higher risks) with unspecified pneumonia^6^ and incident lower respiratory infection.^24^ In CKB, we found no evidence of association with upper respiratory infection, but significant positive association with lower respiratory infection, adding further evidence on the potential harm of NO_2_ on the respiratory system.

Additionally, we found supra-linear positive associations between long-term NO_2_ exposure and musculoskeletal disease. The only relevant cohort analyses were based on the UK Biobank,^6^, ^25^, ^26^ with suggestive evidence of weak positive associations with total musculoskeletal disease (HR 1·007 [95% CI 1·006–1·009]) and several specific endpoints (eg, other intervertebral disc disorders and dorsopathies).^25^ However, all three studies examined only a single year (2010) of NO_2_ concentration as exposure, and the study on total musculoskeletal disease mixed outpatient (with only partial coverage in UK Biobank) and inpatient events, which might have diluted the association.^25^ In CKB, there were insufficient incident cases for most previously examined musculoskeletal outcomes, but we found novel linkages with three common diseases (ie, arthrosis, intervertebral disc disorders, and spondylopathies) in Chinese adults, warranting further investigation in other settings. Of note, we found stronger associations in female participants, never-smokers, and non-drinkers than in their counterparts, but the subgroup differences by smoking and drinking diminished when restricting to male participants. Given the predominance of female participants in never-smokers and never-drinkers in CKB, the subgroup differences were likely to be due to sex, with female individuals known to be more vulnerable to musculoskeletal disease due to genetic, immunological, and hormonal factors.^27^

Furthermore, in CKB each 10 μg/m^3^ NO_2_ exposure was associated with a 12–23% higher risk of incidence of mental and behavioural disorders, with the linear association levelling off at greater than 40 μg/m^3^ NO_2_. Although we lacked sufficient case numbers to investigate specific disease endpoints for mental and behavioural disorders, our findings are coherent with the only previous Chinese cohort study, which reported a 58% (95% CI 42–77) higher risk of depression-related hospitalisation per 8·2 μg/m^3^ higher exposure, with the association levelling off at greater than 35 μg/m^3^.^4^ Of note, we found a stronger association in male participants than female participants. This difference could be due to the higher background risks of mental illnesses in female individuals,^28^ whereby the same absolute impact of NO_2_, if any, might result in a smaller relative impact in the group at higher risk.

In the main analyses, NO_2_ showed no clear associations with other diseases. However, in contrast to the overall null associations with infectious diseases, neoplasms, and endocrine, nutritional and metabolic diseases, there were signs of positive associations in people older than 60 years but not among younger participants. This might reflect how older people, with generally poorer immunity, DNA repair capacity, and insulin sensitivity are more susceptible to the adverse effects of NO_2_·^29^, ^30^, ^31^ Also, after excluding participants with diabetes at baseline, there was an indicative inverse association with endocrine, nutritional and metabolic diseases, but this could be a chance finding. Nervous system diseases were weakly positively associated with NO_2_ in the main model, but this attenuated to the null in the main + PM_2·5_ model. Because long-term NO_2_ exposure has been associated with neuroinflammation,^32^ further studies are needed to clarify the association. For eye and adnexa diseases and digestive diseases, although the linear HR estimates showed marginal significance for inverse association, the exposure–response curves suggest these to be statistical artifacts.

In urban settings with motor traffic as a dominant source of air pollution, NO_2_ and PM_2·5_ levels are often strongly correlated.^1^ This poses long-standing challenges to tease out the independent associations of the two pollutants with disease outcomes, with many previous studies adopting only single-pollutant models.^33^ We used two-pollutant models to explore the issue, and focused on the consistent associations across models. However, the considerable collinearity of NO_2_ and PM_2·5_ could lead to effect transfer, whereby the association with one pollutant is transferred to another correlated pollutant in the same model, with unclear direction and size of transfer. This effect could partly explain the changes in associations with several outcomes between the main and main + PM_2·5_ models, and further studies using more sophisticated methods are needed to clarify this.

The biological mechanisms underlying the associations between NO_2_ and the range of diseases observed in this study are not well understood. However, NO_2_ is a reactive gas that can dissolve in the respiratory epithelial lining fluid, and produce reactive nitrogen and oxygen species and oxidised lipid molecules, thus increasing local and systemic inflammation and oxidative stress.^34^ These factors predispose to impaired respiratory immunity and increased risk of atherosclerosis and hypertension, which are major risk factors of cardiorespiratory diseases.^35^ Systemic inflammation might also trigger autoinflammatory and autoimmune responses that are linked to increased risk of musculoskeletal diseases.^36^ NO_2_ has also been associated with impaired hormonal regulation and endocrine disruption, which might explain the particularly increased risk of musculoskeletal diseases and mental and behavioural disorders in female individuals (because female hormones play a vital role in bone health) and endocrine diseases in older people.^1^

Although the latest GBD estimation for NO_2_ only considered asthma, WHO tightened the air quality guideline for annual average NO_2_ from 40 μg/m^3^ to 10 μg/m^3^ in 2021.^7^ Our findings on the potentially extensive impact of long-term NO_2_ exposure support the ambitious benchmark for stronger policy action, particularly on controlling traffic-related air pollution, most likely by establishing low-emission zones, tightening vehicular emission standards, and promoting clean energy.^1^, ^37^ For future research, larger-scale cohort studies with more diverse exposure patterns and longer follow-up in China and other LMICs are needed to investigate specific diseases beyond the cardiorespiratory systems. There remain major knowledge gaps on the contribution of household sources (eg, gas-fire cooking) to personal NO_2_ exposure, but lessons can be drawn from existing studies that have directly measured personal and household PM_2·5_ within subsets of large-scale cohorts.^38^, ^39^

The key strengths of this study lie in the well validated, high-resolution ambient NO_2_ exposure data, the systematic investigation of a wide spectrum of disease incidence outcomes, the stringent analysis focusing on robust findings across multiple analyses, and the robust null association with a negative control endpoint. However, some limitations warrant discussion. First, despite the large case numbers for the aggregated endpoints, we have only identified 12 specific diseases with reasonably large numbers to enable reliable investigation, given the relatively low exposure heterogeneity within each of the ten localities. Further analysis with longer follow-up should be conducted to verify and expand the present investigation. Second, due to data limitations, we could only examine ambient instead of personal NO_2_ exposure, which could be affected by household sources (eg, gas-fire cooking), although these have been partially accounted for by adjusting for household fuel use. Our focus on ambient NO_2_ would have more direct implications on traffic-related exposure, which could be more readily modified with policy actions. Similarly, despite the use of an advanced spatiotemporal model for NO_2_ assessment, it omits local variations of exposure at street level, and we collected no data on participants’ space-time-activity patterns, so Berkson type exposure misclassification could have occurred, which would lower the precision of the HR estimates.^40^ Like most previous studies, the uncertainty in exposure assessment was not propagated to the prospective analyses due to the limitation of the frequentist methods used. Third, despite the extensive adjustments, residual confounding from certain factors (eg, socioeconomic status, psychological stress, other less studied co-pollutants, and societal-level factors) is plausible. Furthermore, because most covariates were assessed via questionnaire or statistical models (for PM_2·5_ and O_3_), the imperfect measurements might leave residual confounding. Importantly, the observational nature of our study prevented us from establishing causality, and our findings must be further explored in other large prospective cohorts in LMICs. Fourth, CKB was not designed to be representative of Chinese middle-aged adults, but the large sample size and the diversity of the cohort could provide adequate heterogeneity to evaluate epidemiological associations, and the findings could be reasonably generalised to similar populations, although the actual effect estimates might vary.^41^

In conclusion, in a large Chinese cohort, long-term ambient NO_2_ exposure was robustly associated with higher risks of incidence of cardiovascular, respiratory, and musculoskeletal diseases, and there was indicative evidence of associations with mental and behaviour disorders, but further disease-specific investigations are required to clarify these, especially for the effect of PM_2·5_ as a co-pollutant. It is possible that NO_2_ has a much more extensive disease impact than what has previously been captured by GBD, which attributed only childhood asthma to NO_2_ exposure.^2^ The largely linear or supra-linear associations with a wide range of disease outcomes also support the recent tightening of the WHO air quality guideline level for NO_2_, which underscores the pressing need for stronger emission control measures in China and elsewhere.^7^

### China Kadoorie Biobank Study Group

### Contributors

### Data sharing

The CKB is a global resource for the investigation of lifestyle, environmental, blood biochemical, and genetic factors as determinants of common diseases. The CKB Study Group is committed to making the cohort data available to the scientific community in China, the UK, and worldwide to advance knowledge about the causes, prevention, and treatment of disease. For detailed information on what data are currently available to open access users and how to apply for them, visit: https://www.ckbiobank.org/data-access. Researchers who are interested in obtaining the raw data from the CKB study that underlies this paper should contact ckbaccess@ndph.ox.ac.uk. A research proposal will be requested to ensure that any analysis is performed by bona fide researchers and—where data are not currently available to open access researchers—is restricted to the topic covered in this paper.

## Declaration of interests

We declare no competing interests.

## References

[1] HEI Panel on the Health Effects of Long-Term Exposure to Traffic-Related Air Pollution (2022).

[2] GBD 2021 Risk Factors Collaborators (2024). Global burden and strength of evidence for 88 risk factors in 204 countries and 811 subnational locations, 1990–2021: a systematic analysis for the Global Burden of Disease Study 2021. Lancet.

[3] Chen X, Qi L, Li S, Duan X (2024). Long-term NO_2_ exposure and mortality: a comprehensive meta-analysis. Environ Pollut.

[4] Wei F, Yu Z, Zhang X (2022). Long-term exposure to ambient air pollution and incidence of depression: a population-based cohort study in China. Sci Total Environ.

[5] Wang K, Yuan Y, Wang Q (2023). Incident risk and burden of cardiovascular diseases attributable to long-term NO_2_ exposure in Chinese adults. Environ Int.

[6] Hegelund ER, Mehta AJ, Andersen ZJ (2024). Air pollution and human health: a phenome-wide association study. BMJ Open.

[7] WHO (2021).

[8] Wang Y, Luo S, Wei J (2023). Ambient NO_2_ exposure hinders long-term survival of Chinese middle-aged and older adults. Sci Total Environ.

[9] Wright N, Newell K, Chan KH (2023). Long-term ambient air pollution exposure and cardio-respiratory disease in China: findings from a prospective cohort study. Environ Health.

[10] Chen Z, Chen J, Collins R (2011). China Kadoorie Biobank of 0.5 million people: survey methods, baseline characteristics and long-term follow-up. Int J Epidemiol.

[11] Liu C, Chan KH, Lv J (2022). Long-term exposure to ambient fine particulate matter and incidence of major cardiovascular diseases: a prospective study of 0.5 million adults in China. Environ Sci Technol.

[12] Li X, Wang P, Wang W (2023). Mortality burden due to ambient nitrogen dioxide pollution in China: application of high-resolution models. Environ Int.

[13] Ying Q, Li J, Kota SH (2015). Significant contributions of isoprene to summertime secondary organic aerosol in eastern United States. Environ Sci Technol.

[14] Meng X, Liu C, Zhang L (2021). Estimating PM_2.5_ concentrations in northeastern China with full spatiotemporal coverage, 2005–2016. Remote Sens Environ.

[15] Meng X, Wang W, Shi S (2022). Evaluating the spatiotemporal ozone characteristics with high-resolution predictions in mainland China, 2013–2019. Environ Pollut.

[16] Yang L, Chen Z, Chen Z (2020). Population biobank studies: a practical guide.

[17] Murray CJL, Aravkin AY, Zheng P (2020). Global burden of 87 risk factors in 204 countries and territories, 1990–2019: a systematic analysis for the Global Burden of Disease Study 2019. Lancet.

[18] Liu S, Jørgensen JT, Ljungman P (2021). Long-term exposure to low-level air pollution and incidence of asthma: the ELAPSE project. Eur Respir J.

[19] Turnbull I, Clarke R, Wright N (2022). Diagnostic accuracy of major stroke types in Chinese adults: a clinical adjudication study involving 40,000 stroke cases. Lancet Reg Health West Pac.

[20] Verhoeven JI, Allach Y, Vaartjes ICH, Klijn CJM, de Leeuw F-E (2021). Ambient air pollution and the risk of ischaemic and haemorrhagic stroke. Lancet Planet Health.

[21] Amini H, Dehlendorff C, Lim Y-H (2020). Long-term exposure to air pollution and stroke incidence: a Danish Nurse Cohort study. Environ Int.

[22] Liu S, Lim YH, Pedersen M (2021). Long-term air pollution and road traffic noise exposure and COPD: the Danish Nurse Cohort. Eur Respir J.

[23] Chan KH, Kurmi OP, Bennett DA (2019). Solid fuel use and risks of respiratory diseases: a cohort study of 280,000 Chinese never-smokers. Am J Respir Crit Care Med.

[24] Zhang J, Lim YH, So R (2024). Long-term exposure to air pollution and risk of acute lower respiratory infections in the Danish Nurse Cohort. Ann Am Thorac Soc.

[25] Cheng B, Pan C, Cai Q (2024). Long-term ambient air pollution and the risk of musculoskeletal diseases: a prospective cohort study. J Hazard Mater.

[26] Zhang J, Fang XY, Wu J (2023). Association of combined exposure to ambient air pollutants, genetic risk, and incident rheumatoid arthritis: a prospective cohort study in the UK Biobank. Environ Health Perspect.

[27] Overstreet DS, Strath LJ, Jordan M (2023). A brief overview: sex differences in prevalent chronic musculoskeletal conditions. Int J Environ Res Public Health.

[28] Bai R, Dong W, Peng Q, Bai Z (2022). Trends in depression incidence in China, 1990–2019. J Affect Disord.

[29] Cisneros B, García-Aguirre I, Unzueta J (2022). Immune system modulation in aging: molecular mechanisms and therapeutic targets. Front Immunol.

[30] Maynard S, Fang EF, Scheibye-Knudsen M, Croteau DL, Bohr VA (2015). DNA damage, DNA repair, aging, and neurodegeneration. Cold Spring Harb Perspect Med.

[31] Shou J, Chen P-J, Xiao W-H (2020). Mechanism of increased risk of insulin resistance in aging skeletal muscle. Diabetol Metab Syndr.

[32] McLachlan J, Cox SR, Pearce J, Valdés Hernández MdC (2023). Long-term exposure to air pollution and cognitive function in older adults: a systematic review and meta-analysis. Front Environ Health.

[33] Committee on the Medical Effects of Air Pollutants (2018).

[34] Sandström T (1995). Respiratory effects of air pollutants: experimental studies in humans. Eur Respir J.

[35] Zhao C-N, Xu Z, Wu G-C (2019). Emerging role of air pollution in autoimmune diseases. Autoimmun Rev.

[36] Gallo J, Raska M, Kriegova E, Goodman SB (2017). Inflammation and its resolution and the musculoskeletal system. J Orthop Translat.

[37] Chamberlain RC, Fecht D, Davies B, Laverty AA (2023). Health effects of low emission and congestion charging zones: a systematic review. Lancet Public Health.

[38] Shupler M, Hystad P, Birch A (2020). Household and personal air pollution exposure measurements from 120 communities in eight countries: results from the PURE-AIR study. Lancet Planet Health.

[39] Chan KH, Xia X, Liu C (2023). Characterising personal, household, and community PM_2.5_ exposure in one urban and two rural communities in China. Sci Total Environ.

[40] Samet JM, Dominici F, Zeger SL, Schwartz J, Dockery DW (2000).

[41] Rothman KJ, Gallacher JE, Hatch EE (2013). Why representativeness should be avoided. Int J Epidemiol.

