# Supplementary material for: Associations of long-term nitrogen dioxide exposure with a wide spectrum of diseases: a prospective cohort study of 0·5 million Chinese adults
Source: Lancet Public Health. 2024 Dec 4;9(12):e1047–58. doi: 10.1016/S2468-2667(24)00264-0 (PMC11626078; doi:10.1016/S2468-2667(24)00264-0)
Supplement: Supplementary appendix [file mmc1.pdf]

THE LANCET
Public Health

Supplementary appendix

This appendix formed part of the original submission and has been peer reviewed. We post it as supplied by the authors.

Supplement to: Xia X, Meng X, Liu C, et al. Associations of long-term nitrogen dioxide exposure with a wide spectrum of diseases: a prospective cohort study of 0·5 million Chinese adults. *Lancet Public Health* 2024; **9:** e1047–58.

Supplementary Appendix

The associations of long-term NO_2 exposure with a wide spectrum of diseases: a prospective cohort study of 0.5 million Chinese adults

Xi Xia, PhD[1,2,3,4*], Xia Meng, PhD[5*], Cong Liu, PhD[5*], Yi Guo, MPhil[5], Xinyue Li, MPhil[5], Yue Niu, PhD[5], Kin Bong Hubert Lam, PhD[3], Neil Wright, PhD[3], Christiana Kartsonaki, DPhil[3], Yiping Chen, DPhil[3], Ling Yang, PhD[3], Huaidong Du, PhD[3], Canqing Yu, PhD[6,7,8], Dianjianyi Sun, PhD[6,7,8], Jun Lv, PhD[6,7,8], Junshi Chen, MD[9], Xiaoming Yang, PhD[3], Ruqin Gao, MPhil[10], Shaowei Wu, PhD[1,2†], Haidong Kan, PhD[5,11†], Ka Hung Chan, DPhil[3†], Liming Li, PhD[6,7,8‡], Zhengming Chen, DPhil[3‡] on behalf of the China Kadoorie Biobank Study Group[#]

1. Department of Occupational and Environmental Health, School of Public Health, Xi'an Jiaotong University Health Science Center, Xi'an, China
2. Key Laboratory of Environment and Genes Related to Diseases, Ministry of Education, Xi'an, China
3. Clinical Trial Service Unit and Epidemiological Studies Unit, Nuffield Department of Population Health, University of Oxford, Oxford, UK
4. School of Public Health, Shaanxi University of Chinese Medicine, Xi'an, China
5. School of Public Health, Key Lab of Public Health Safety of the Ministry of Education and NHC Key Lab of Health Technology Assessment, Fudan University, Shanghai, China
6. Department of Epidemiology and Biostatistics, School of Public Health, Peking University Health Science Center, Beijing, China
7. Peking University Center for Public Health and Epidemic Preparedness & Response, Beijing, China
8. Key Laboratory of Epidemiology of Major Diseases (Peking University), Ministry of Education, Beijing, China
9. China National Center For Food Safety Risk Assessment, Beijing, China
10. Qingdao Center for disease Control and Prevention, Qingdao, China
11. Children's Hospital of Fudan university, National Center for Children's Health, Shanghai, China

*Joint first authors; †Joint corresponding authors; ‡Joint Senior authors; # Members of the CKB Collaborative Group are shown at the end of the manuscript

Addresses for correspondence

Ka Hung Chan: Big Data Institute, Old Road Campus, Roosevelt Drive, OX3 7LF; email: peter.chan.oxford@gmail.com;
Haidong Kan: School of Public Health, Key Lab of Public Health Safety of the ministry of Education and NHC Key Lab of Health Technology Assessment, Fudan University, Shanghai 200032, China; email: kanh@fudan.edu.cn;
Shaowei Wu: Department of Occupational and Environmental Health, School of Public Health, Xi'an Jiaotong University Health Science Center, 76 Yanta West Road, Yanta District, Xi'an, Shaanxi 710061, China; email: shaowei_wu@xjtu.edu.cn

Contents

Supplementary Methods I - Estimation of $PM_{2.5}$ and O_3 exposure 4

Supplementary Methods II – Sources of air pollution considered in the NO_2 exposure model 5

Supplementary Methods III – Outcome ascertainment and disease adjudication.......... 7

Supplementary Methods IV – Rationale of examining composite incidence endpoints 8

Supplementary Methods V – Covariates selection and parametrisation 9

Supplementary Methods VI – Detailed rationale and limitations of two-pollutant models 11

Supplementary Methods VII – Detailed rationale to select the 12 specific disease endpoints.... 12

eTable 1. ICD-10 chapters included in the main analyses and the respective incident event numbers 13

eTable 2. Number of incident events for specific outcomes selected for further analysis 14

eTable 3. Pearson and Spearman's rank correlation between NO_2, $PM_{2.5}$, and O_3 by study areas 15

eTable 4. Cochrane's Q test for the difference between main model and two-pollutant models. 16

eTable 5. Cochrane's Q test for the difference between main model and sensitivity models 17

eTable 6. Estimated population attributable fraction for disease outcomes showing significant associations with NO_2 exposure across all main models and in sensitivity analyses.......... 18

eFigure 1. Study sites of the China Kadoorie Biobank 19

eFigure 2. Flow chart of exclusion of participants 20

eFigure 3. Hazard ratios (HR) and 95% confidence intervals (CIs) for incidence of 14 ICD-10 chapter-based endpoints per 10 μg/m^3 higher annual NO_2 exposure in the two pollutant models: a) main + $PM_{2.5}$ model and b) main + O_3 model 21

eFigure 4. Hazard ratios (HR) and 95% confidence intervals (CIs) for incidence of 12 specific causes per 10 μg/m^3 higher annual NO_2 exposure in the two-pollutant models: a) main + $PM_{2.5}$ model and b) main + O_3 model The solid boxes represent HRs, with the size inversely proportional to the variance of the logarithm of the HR, and the horizontal lines represent 95% CIs. All models were stratified by age-at-risk (in 1-year scale), ten study areas, and sex, and were adjusted for education, household income, smoking status, alcohol drinking, cooking fuel type, heating fuel type, self-rated health status, body mass index (BMI), physical activity level, temperature and relative humidity. The models fit from the 1th to 99th percentiles of NO_2 concentration. Abbreviations: CI, confidence interval; HR, hazard ratio. 22

eFigure 5. Exposure-response relationships of annual ambient NO_2 exposure with disease incidence of 14 ICD-10 chapter-based endpoints in the main models, stratified by age.......... 23

eFigure 6. Hazard ratios (HR) and 95% confidence intervals (CIs) for incidence of 14 ICD-10 chapter-based endpoints per 10 μg/m^3 higher annual ambient NO_2 exposure in the main models, stratified by age 24

eFigure 7. Exposure-response relationships of annual ambient NO_2 exposure with incidence of 14 ICD-10 chapter-based endpoints in the main models, stratified by sex 25

eFigure 8. Hazard ratios (HR) and 95% confidence intervals (CIs) for incidence of 14 ICD-10 chapter-based endpoints per 10 μg/m^3 higher annual ambient NO_2 exposure in the main models, stratified by sex 26

eFigure 9. Exposure-response relationships of annual ambient NO_2 exposure with incidence of 14 ICD-10 chapter-based endpoints in the main models, stratified by smoking status 27

eFigure 10. Hazard ratios (HR) and 95% confidence intervals (CIs) for incidence of 14 ICD-10 chapter-based endpoints per 10 μg/m^3 higher annual ambient NO_2 exposure in the main models, stratified by smoking status 28

eFigure 11. Exposure-response relationships of annual ambient NO_2 exposure with incidence of 14 ICD-10 chapter-based endpoints in the main models, stratified by physical activity levels 29

eFigure 12. Hazard ratios (HR) and 95% confidence intervals (CIs) for incidence of 14 ICD-10 chapter-based endpoints per 10 μg/m^3 higher annual ambient NO_2 exposure in the main models, stratified by physical activity levels 30

eFigure 13. Exposure-response relationships of annual ambient NO_2 exposure with incidence across 14 ICD-10 chapter-based endpoints in the main models, stratified by drinking status 31

eFigure 14. Hazard ratios (HR) and 95% confidence intervals (CIs) for incidence of 14 ICD-10 chapter-based endpoints per 10 μg/m^3 higher annual ambient NO_2 exposure in the main models, stratified by drinking status 32

eFigure 15. Exposure-response relationships of annual ambient NO_2 exposure with incidence of musculoskeletal and mental & behavioural disease in the main models stratified by smoking and drinking status 33

eFigure 16. Hazard ratios (HR) and 95% confidence intervals (CIs) for incidence of 14 ICD-10 chapter-based endpoints per 10 μg/m_3 higher annual ambient NO_2 exposure in the main models and main + $PM_{2.5}$ two-pollutant models after excluding participants with relevant disease incidence during the first three years of follow-up 34

eFigure 17. Hazard ratios (HR) and 95% confidence intervals (CIs) for incidence of eight ICD-10 chapter-based endpoints per 10 μg/m^3 higher annual ambient NO_2 exposure in the main models and main + $PM_{2.5}$ two-pollutant models after excluding participants with relevant prior medical history at baseline 35

eFigure 18. Lag patterns for associations (hazard ratios) of long-term NO_2 exposure with incidence of 14 ICD-10 chapter-based endpoints in the main models 36

Supplementary Methods I – Assessment of air pollution exposure

The methodological and validation details of the NO_2 model has been published elsewhere.[14] To supplement the main text, the meteorological parameter of the NO_2 model included air temperature, relative humidity, total precipitation, wind speed, surface air pressure, total cloud fraction, and planetary boundary layer height. The other ancillary variables included elevation, population density, road networks, and normalized difference vegetation index.

For the residential $PM_{2.5}$ and O_3 exposure models, a gap-filling approach was developed to link ground-level $PM_{2.5}$ measurements and a list of predictors (i.e., simulated $PM_{2.5}$ concentrations from MERRA-2 [Modern-Era Retrospective Analysis for Research and Applications, Version 2], aerosol optical depth product, meteorological parameters, land use, population density, and visibility data).[13,16,17] The cross-validated R^2 between daily $PM_{2.5}$ measurements and predictions was 0.81 (RMSE, 18.5 μg/m³). For O_3 estimation model, the ground-level maximum daily 8 h average (MDA8) O_3 measurements was dependent variable and spatiotemporal predictors were independent variables (i.e., community Multiscale Air Quality simulations, meteorological parameters, elevation, road networks, and population data). The cross-validated R^2 between monthly O_3 measurements and predictions was 0.83 (RMSE, 14.46 μg/m³), indicating a relatively high accuracy of prediction.

Supplementary Methods II – Sources of air pollution considered in the NO_2 exposure model

As per many existing studies of similar kind, the NO_2 exposure data came from nationwide spatiotemporal models developed and described separately.[1] The models incorporated a wide range of exposure predictors, including satellite remote sensing data (NO_2 vertical column density), simulated NO_2 concentrations from community multiscale air quality (CMAQ) model, meteorological data, elevation, population, road networks, and Normalized Difference Vegetation Index (NDVI). According to the nationwide distribution of NO_2 prediction, we could infer that anthropogenic emissions, e.g., traffic emissions would have played leading roles in determining the intra- and inter-area heterogeneity of NO_2 levels, since the spatial distribution of predicted NO_2 concentrations (Figure SMII.1) were highly consistent with that of road network (Figure SMII.2A) and population density (Figure SMII.2B) reported previously[1] and as shown in the figures below.

Figure SMII.1: Spatial distribution of annual mean NO_2 concentrations at 1 km x 1 km spatial resolution in 2019 (adopted from Li et al. 2023[1]).

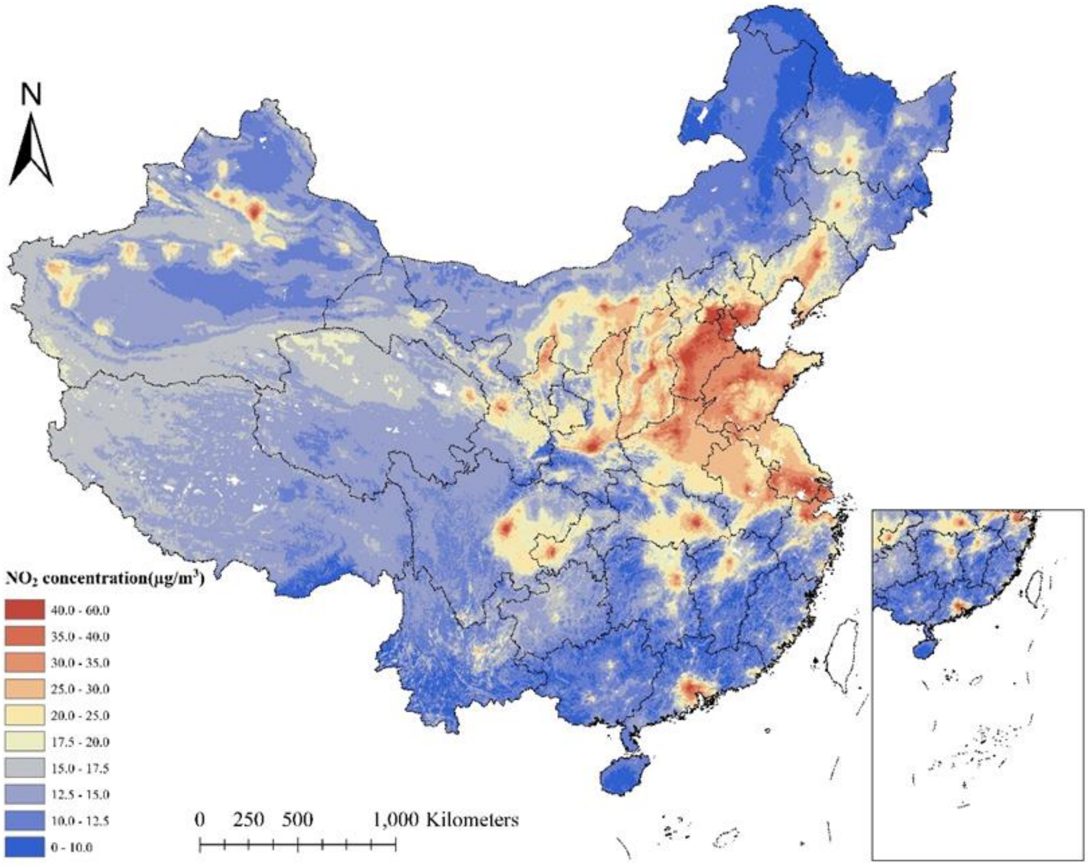

Figure SMII.2: Spatial distribution of of the total length of all raod types and population size within 1 km grids in mainland China in 2019 (adopted from Li et al. 2023[1]).

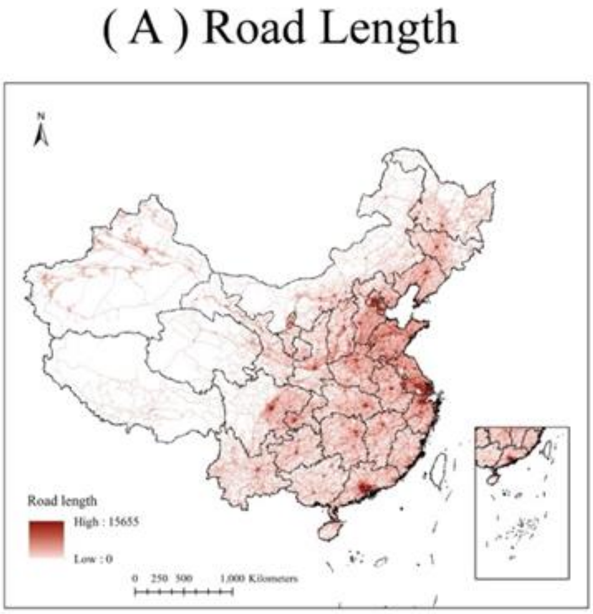

Supplementary Methods III – Outcome ascertainment and disease adjudication

After the baseline survey, participants were continuously followed up for death and any episodes of hospitalisation via electronic linkages (using unique personal identification numbers, name, date of birth, and sex) to death and disease registries and national health insurance databases. Overall, the outcome ascertainment systems covered >96% of all mortality and hospitalisation events, according to internal records cross-validated across multiple healthcare databases, death registries, and local police and administrative records).[2] All events were coded according to the International Classification of Disease and Injuries, 10th Revision (ICD-10) by trained staff blinded to participants' baseline information. For cases where the electronic medical records included complex textual disease descriptions, a bespoke IT software developed with clinicians was applied to automatically capture relevant text and assign correct ICD-10 codes.[3] Based on the bespoke disease outcome verification and adjudication systems in CKB, detailed medical records from over 113,000 hospitalised participants had been retrieved for standardised adjudication on the accuracy of diagnosis of major causes of disease burden, including cardiovascular disease, cancer, chronic kidney disease, and chronic obstructive pulmonary disease, with reporting accuracy rates of 86-94%.[4]

Since death can act as a competing risk for disease events, by censoring participants at death from causes other than the one being analysed, we estimated cause-specific HRs that compare event rates in surviving participants who are free of the event of interest, thus providing a valid assessment of the aetiological questions of interest.[19]

Supplementary Methods IV – Rationale of examining composite incidence endpoints

For many major causes of disease burden, substantial proportions of the first events are fatal in the study population (e.g., 40% of acute myocardial infraction in CKB); whereas for milder conditions most first events are non-fatal hospital admissions (e.g., 98.5% of digestive diseases in CKB). By examining composite endpoints capturing the earliest recorded event per participant, through survival analysis we can most appropriately assess whether the risk exposure was associated with higher rates of the disease outcome or, essentially, shorter time to the emergence of first events. If the risk exposure does cause disease development, whether such first events were fatal or non-fatal should not alter the answer to the aetiological question of interest. On the contrary, while traditionally many earlier cohort studies examined only mortality endpoints due to data availability and quality issues (e.g., all-cause mortality data tend to be more reliably documented, but the attributed cause still suffer from outcome misclassification like hospital admission), studying mortality alone would suffer from a greater extent of confounding from unaccounted risk factors between disease emergence and mortality (e.g. treatment quality, access to care).[5]

Supplementary Methods V – Covariates selection and parametrisation

The covariates in the main analyses were selected based on prior knowledge and the observed relationships with NO_2 exposure. In particular, the Cox models were stratified by age-at-risk (in 1-year scale), ten study areas, and sex (where applicable) because these have been determined as fundamentally important factors where the baseline hazards for disease outcomes likely differ across strata. The 1-year scale of age-at-risk was selected to match the choice of time-varying annual average NO_2 exposure, so the confounding effects of age-at-risk (which increases over time) would be fully accounted for (as opposed to adjusting for baseline age in simpler models).[6] Stratification by the ten study areas ensure adjustment for unmeasured area-level confounding, such as regional socioeconomic status, local culture (e.g. diet, specific habits), and background disease patterns, all of which are vastly diverse across areas. While this approach means that the models do not make use of the exposure contrasts between study areas (which would reduce the statistical power of the analysis), it is essential to address regional-level confounding and differential baseline hazards by area. Other factors were adjusted to control for confounding effects from socioeconomic status (education [no formal education, primary, middle, high-school or above] and household income [<20,000, 20,000-34,999, ≥35,000 Yuan/year]), lifestyle (smoking [never-regular, occasional, ex-regular, current-regular], alcohol drinking [never-regular, ex-regular, occasional or seasonal, monthly, reduced intake, weekly], physical activity [continuous, in MET-hr/day]), anthropometric and health (BMI [continuous], self-rated health status [poor, fair, good, excellent]), and environmental factors (cooking and heating fuel exposure [never-regular cooking/ heating, always clean fuels, solid to clean fuels, always solid fuels, others], annual average temperature [continuous, °C], and relative humidity [continuous, %]).

Physical activity was assessed following a validated questionnaire-based approach described in details previously.[7,8] Briefly, a questionnaire assessed the intensity, frequency,

time spent on occupational tasks, commuting, household tasks, and leisure time activities, and the corresponding total MET-hr/day was calculated following the 2011 compendium of physical activities.[9] The adjustment for self-rated health aims to account for potential confounding from unmeasured or un-reported prevalent disease or subclinical conditions, as well as social deprivation or other unmeasured factors (e.g., noise, current mental health status) that are correlated with self-rated health, NO_2 exposure, and subsequent disease risks. Clean fuels refer to gas, electricity, or district heating (for heating only); whereas solid fuels refers to wood, charcoal, or coal.

Supplementary Methods VI – Detailed rationale and limitations of two-pollutant models

While the primary focus of the present study was to generate novel evidence on the associations of long-term NO_2 exposure with a wide spectrum of diseases in China, it is important to note that multiple air pollutants are often correlated with each other, and may confound the associations observed in single-pollutant models applied in the main analyses. To explore for the potential confounding from key co-pollutants ($PM_{2.5}$ and O_3), we fitted two-pollutant models by introducing annual averages of ambient $PM_{2.5}$ (main+$PM_{2.5}$ model) or O_3 (main+O_3 model) into the main Cox models. This is one of the most commonly applied approaches to explore for potential confounding from co-pollutants in previous studies.[10,11] However, the outputs from two-pollutant models may be difficult to interpret when the co-pollutants are strongly correlated (i.e., collinear), which is the case for NO_2 and $PM_{2.5}$ in most studies including our own (**eTable 3**). While in most previous studies the associations found in the single- and two-pollutant models tended to be consistent or somewhat stronger, it is important to note the possibility of “effect transfer”, whereby the association with one pollutant can be “transferred” to the co-pollutant in unclear size and direction. For the purpose of this study, we followed recommendations from the UK Committee on the Medical Effects of Air Pollutants (COMEAP)[10,11] to report findings from both single- and two-pollutant models, but we took a cautious approach to consider the associations that are consistent across models reliable, and the inconsistent ones subject to further investigation in dedicated studies. We have assessed the variance inflation in the two-pollutant models by computing variance inflation factors from the design matrix of covariates in each Cox model, and found relatively minor indication of collinearity (variance inflation factor for NO_2 and co-pollutants [$PM_{2.5}$ or O_3] <1.5 in all models).

Supplementary Methods VII – Detailed rationale to select the 12 specific disease endpoints

Our study was designed to systematically examine a wide spectrum of disease outcomes that may be related to long-term NO_2 exposure, so we had no prior determination on the specific diseases to be examined. Despite the large sample size of CKB, our highly stringent, stratified Cox regression models would distribute the cases across strata of age-at-risk (n=50), sex (n=2), and study areas (n=10). Therefore, in order for the Cox models to be stable, we needed adequately large case number to minimise spurious findings. As there is no well-established method for accurate power calculation for stratified, time-varying, multivariable Cox models, we determined based on our prior experience that we would need at least 3000 cases for the analysis to have minimally reasonable power. While all aggregated endpoints by ICD-10 chapters satisfied this criteria, we took an even more cautious approach to identify specific diseases under the three ICD-10 chapters (i.e. cardiovascular, respiratory, and musculoskeletal diseases) that showed robust associations with NO_2 across the main and sensitivity analyses, and we found 12 outcomes also satisfying the case number criteria.

eTable 1. ICD-10 chapters included in the main analyses and the respective incident event numbers

ICD-10 Chapter	ICD-10 code	Incident events
I: Infectious	A00-A99, B00-B99	18,767
II: Neoplasms	C00-C97, D00-D48	47,724
IV: Endocrine, nutritional & metabolic	E00-E90	42,819
V: Mental & behavioural	F00-F99	5,361
VI: Nerve-related	G00-G99	29,810
VII: Eye & adnexa	H00-H59	25,754
VIII: Ear & mastoid process	H60-H95	7,470
IX: Cardiovascular	I00-I99	144,852
X: Respiratory	J00-J99	73,232
XI: Digestive	K00-K93	74,729
XII: Skin & subcutaneous tissue	L00-L99	5,285
XIII: Musculoskeletal	M00-M99	54,409
XIV: Genitourinary	N00-N99	41,334
XIX & XX: External causes conditions	S00-S99, T00-T98, V01-Y98	32,907

Abbreviation: ICD-10, International Classification of Diseases 10th revision.

eTable 2. Number of incident events for specific outcomes selected for further analysis

Specific outcomes	ICD-10 code	Incidence
**IX: Cardiovascular disease**		
Hypertensive disease	I10-I15	47,573
Ischaemic heart disease	I20-I25	53,936
Intracerebral haemorrhage	I61	12,325
Ischaemic stroke	I63	53,336
Other cerebrovascular disease	I60, I62, I64-I69	38,882
**X: Respiratory disease**		
Acute URTI	J00-J06	8,118
Pneumonia & other LRTI	J12-J18, J20-J22	41,805
Chronic lower respiratory disease	J40-J44	25,986
Asthma	J45	3,154
**XIII: Musculoskeletal disease**		
Arthrosis	M15-M19	10,282
Spondylopathies	M45-M49	19,912
Intervertebral disc disorders	M50-M51	21,074

Abbreviations: ICD-10, International Classification of Diseases 10th revision; LRTI, lower respiratory tract infections; URTI, Upper respiratory tract infections.

eTable 3. Pearson and Spearman's rank correlation between NO_2, $PM_{2.5}$, and O_3 by study areas

Study site	Pearson correlation		Spearman correlation	
	NO_2 & $PM_{2.5}$	NO_2 & O_3	NO_2 & $PM_{2.5}$	NO_2 & O_3
Huixian	0.80	-0.02	0.76	-0.13
Suzhou	0.68	-0.52	0.72	-0.36
Tongxiang	0.68	-0.22	0.66	-0.30
Harbin	0.86	-0.91	0.84	-0.85
Qingdao	0.70	-0.92	0.73	-0.90
Pengzhou	0.42	-0.36	0.39	-0.51
Liuyang	0.73	0.06	0.73	0.10
Liuzhou	0.76	-0.63	0.79	-0.66
Maiji	0.76	-0.42	0.85	-0.54
Haikou	0.77	-0.13	0.78	-0.08

eTable 4. Cochrane's Q test for the difference between main model and two-pollutant models

ICD-10 Chapter	Main model vs Main+$PM_{2.5}$	Main model vs Main+O_3
I: Infectious	0.013*	0.994
II: Neoplasms	0.938	0.774
IV: Endocrine, nutritional & metabolic	0.092	0.293
V: Mental & behavioural	0.102	0.466
VI: Nerve-related	0.006*	0.507
VII: Eye & adnexa	0.086	0.441
VIII: Ear & mastoid process	0.000*	0.637
IX: Cardiovascular	0.024*	0.942
X: Respiratory	0.001*	0.977
XI: Digestive	0.000*	0.891
XII: Skin & subcutaneous tissue	0.758	0.841
XIII: Musculoskeletal	0.000*	0.505
XIV: Genitourinary	0.001*	0.978
XIX & XX: External causes conditions	0.511	0.766

Abbreviation: ICD-10, International Classification of Diseases 10th revision.

eTable 5. Cochrane’s Q test for the difference between main model and sensitivity models

ICD-10 Chapter	Main model vs Main remove first 3 years	Main model vs Main excluding prevalent disease at baseline
I: Infectious	0.763	NA
II: Neoplasms	0.733	0.979
IV: Endocrine, nutritional & metabolic	0.707	0.002
V: Mental & behavioural	0.252	0.884
VI: Nerve-related	0.386	NA
VII: Eye & adnexa	0.896	NA
VIII: Ear & mastoid process	0.892	NA
IX: Cardiovascular	0.584	0.533
X: Respiratory	0.804	0.770
XI: Digestive	0.637	0.968
XII: Skin & subcutaneous tissue	0.832	NA
XIII: Musculoskeletal	0.654	0.748
XIV: Genitourinary	0.678	0.744
XIX & XX: External causes conditions	0.694	NA

Abbreviation: ICD-10, International Classification of Diseases 10th revision.

eTable 6. Estimated population attributable fraction for disease outcomes showing significant associations with NO_2 exposure across all main models and in sensitivity analyses

ICD-10 Chapter	PAF (95% CI) %
IX: Cardiovascular	18.3 (16.9-19.7)
X: Respiratory	6.8 (4.5-9.0)
XIII: Musculoskeletal	32.0 (30.1-33.9)

Note: HRs and 95% CIs for per 10 μg/m^3 increase in NO_2 were shown. The annual average concentration <10 μg/m^3 was the reference level for NO_2.
Abbreviations: CI, confidence interval; HR, hazard ratio; PAF, population attributable fraction.

eFigure 1. Study sites of the China Kadoorie Biobank

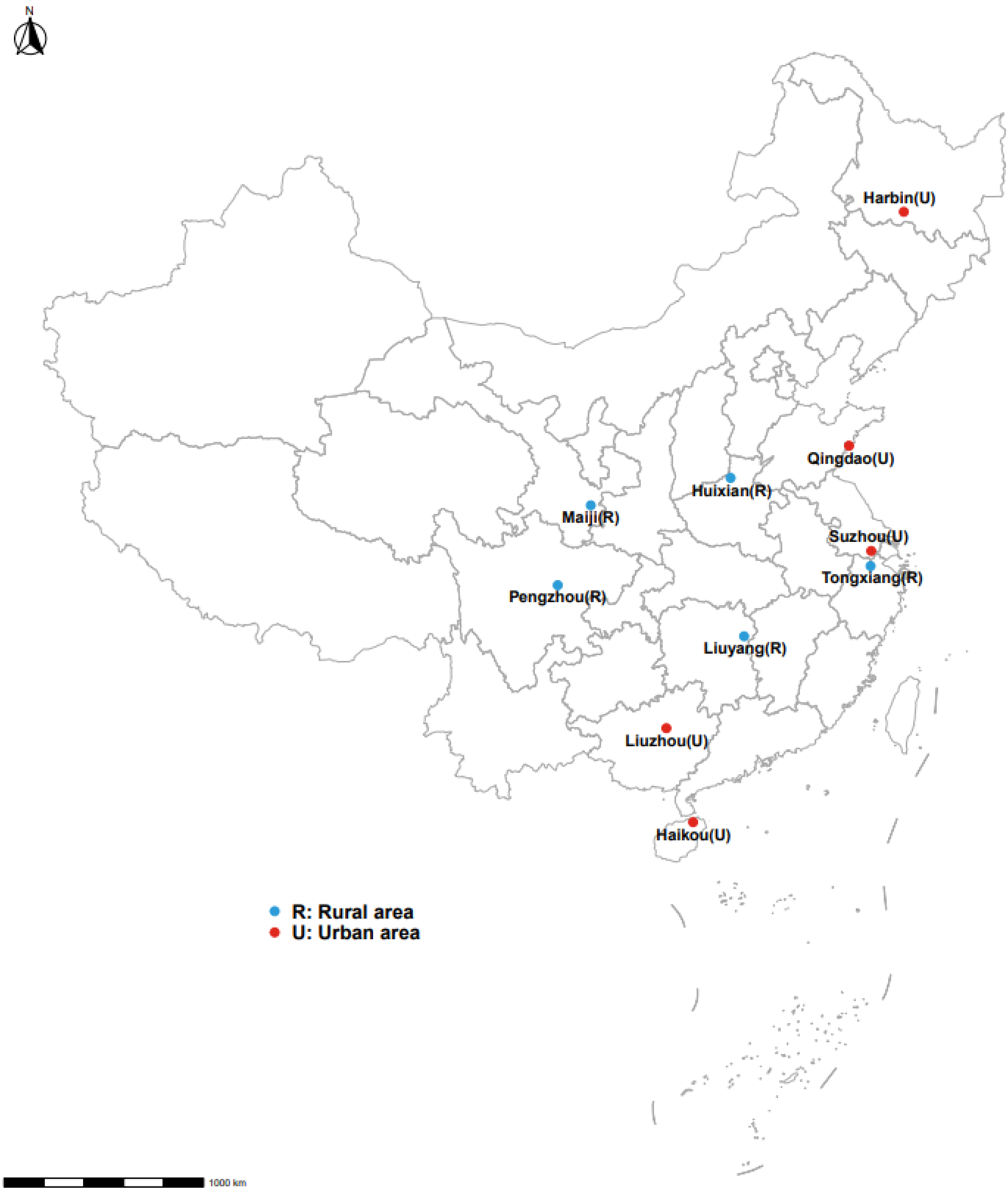

eFigure 2. Flow chart of exclusion of participants

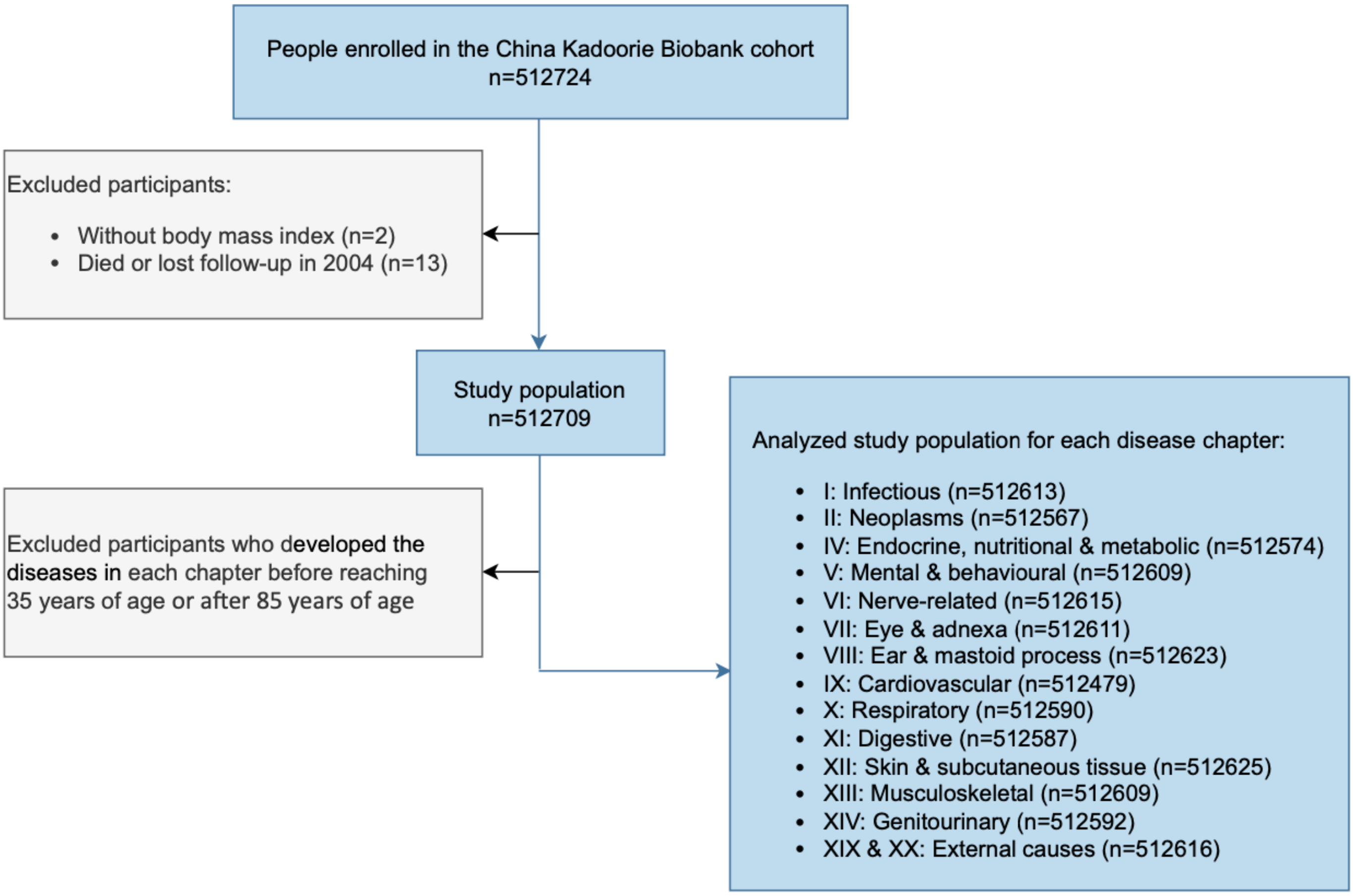

eFigure 3. Hazard ratios (HR) and 95% confidence intervals (CIs) for incidence of 14 ICD-10 chapter-based endpoints per 10 μg/m^3 higher annual NO_2 exposure in the two pollutant models: a) main + $PM_{2.5}$ model and b) main + O_3 model

The solid boxes represent HRs, with the size inversely proportional to the variance of the logarithm of the HR, and the horizontal lines represent 95% CIs. All models were stratified by age-at-risk (in 1-year scale), ten study areas, and sex, and were adjusted for education, household income, smoking status, alcohol drinking, cooking fuel type, heating fuel type, self-rated health status, body mass index (BMI), physical activity level, temperature and relative humidity. The models fit from the 1th to 99th percentiles of NO_2 concentration. Abbreviations: CI, confidence interval; HR, hazard ratio.

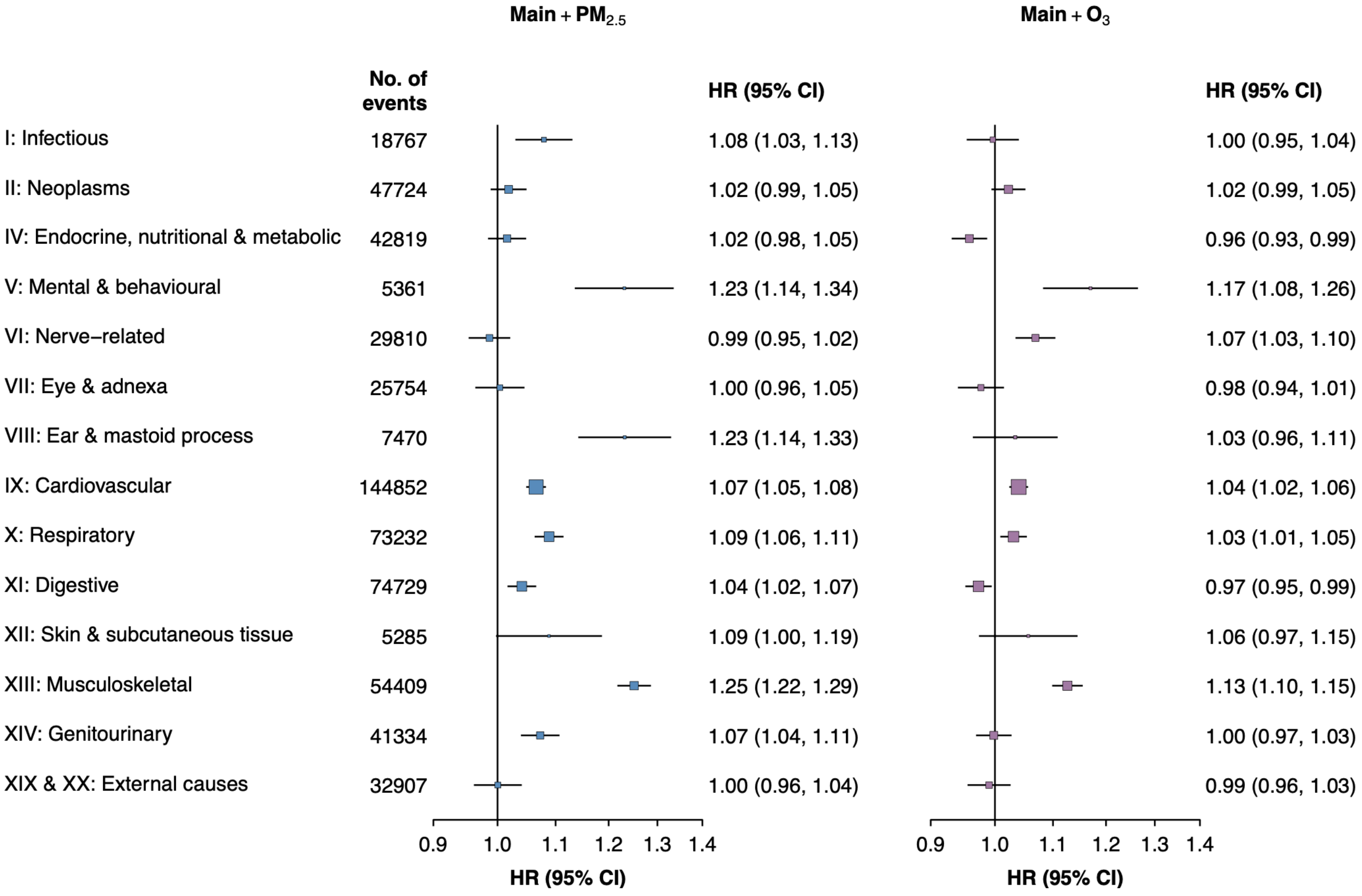

eFigure 4. Hazard ratios (HR) and 95% confidence intervals (CIs) for incidence of 12 specific causes per 10 μg/m^3 higher annual NO_2 exposure in the two-pollutant models: a) main + $PM_{2.5}$ model and b) main + O_3 model

The solid boxes represent HRs, with the size inversely proportional to the variance of the logarithm of the HR, and the horizontal lines represent 95% CIs. All models were stratified by age-at-risk (in 1-year scale), ten study areas, and sex, and were adjusted for education, household income, smoking status, alcohol drinking, cooking fuel type, heating fuel type, self-rated health status, body mass index (BMI), physical activity level, temperature and relative humidity. The models fit from the 1th to 99th percentiles of NO_2 concentration. Abbreviations: CI, confidence interval; HR, hazard ratio.

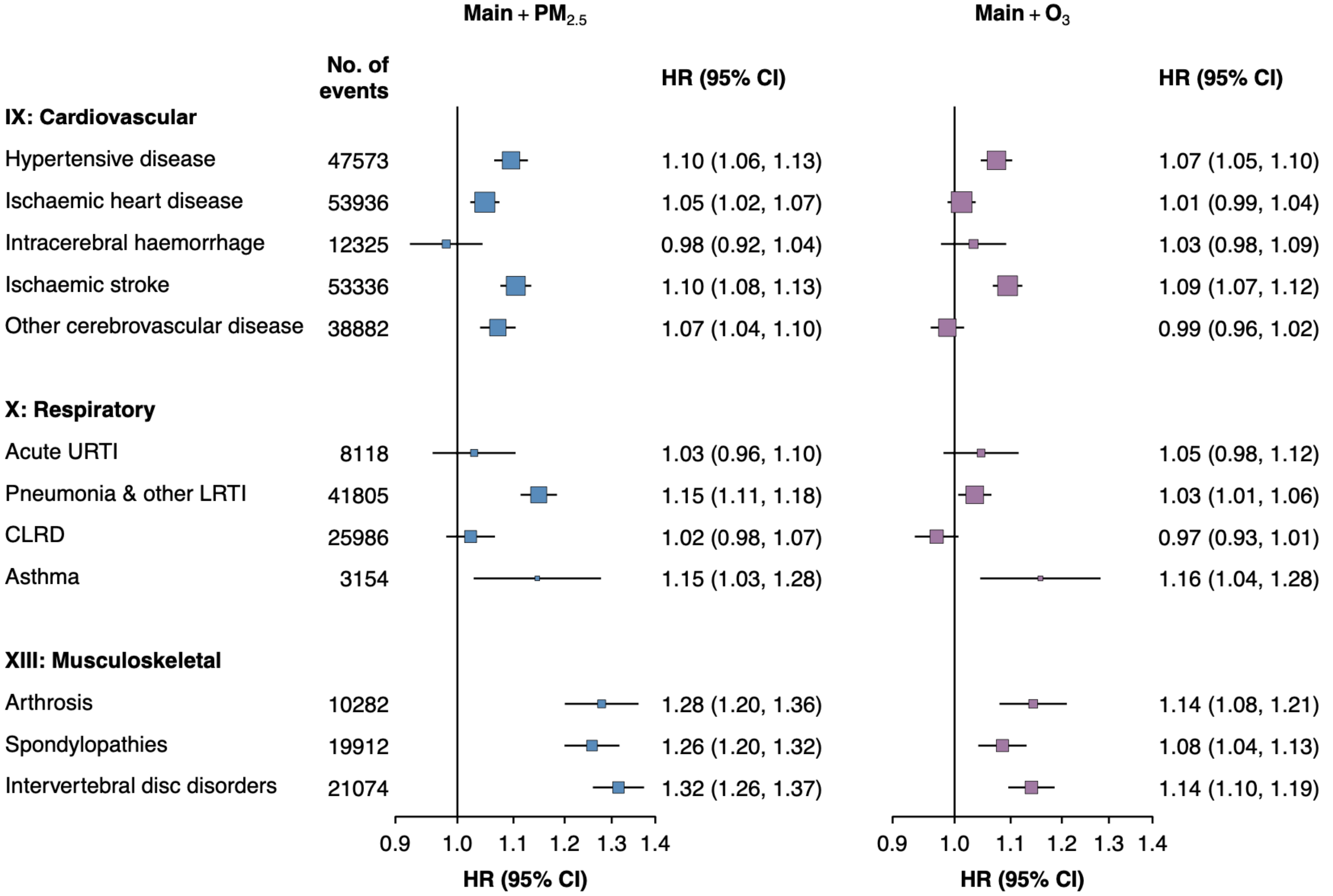

eFigure 5. Exposure-response relationships of annual ambient NO_2 exposure with disease incidence of 14 ICD-10 chapter-based endpoints in the main models stratified by age

Note: Solid line represents HRs and the ribbon represents its 95% CI. All models were stratified by age-at-risk (in 1-year scale), ten study areas, and sex, and were adjusted for education, household income, smoking status, alcohol drinking, cooking fuel type, heating fuel type, self-rated health status, body mass index (BMI), physical activity level, temperature and relative humidity. Abbreviations: CI, confidence interval; HR, hazard ratio.

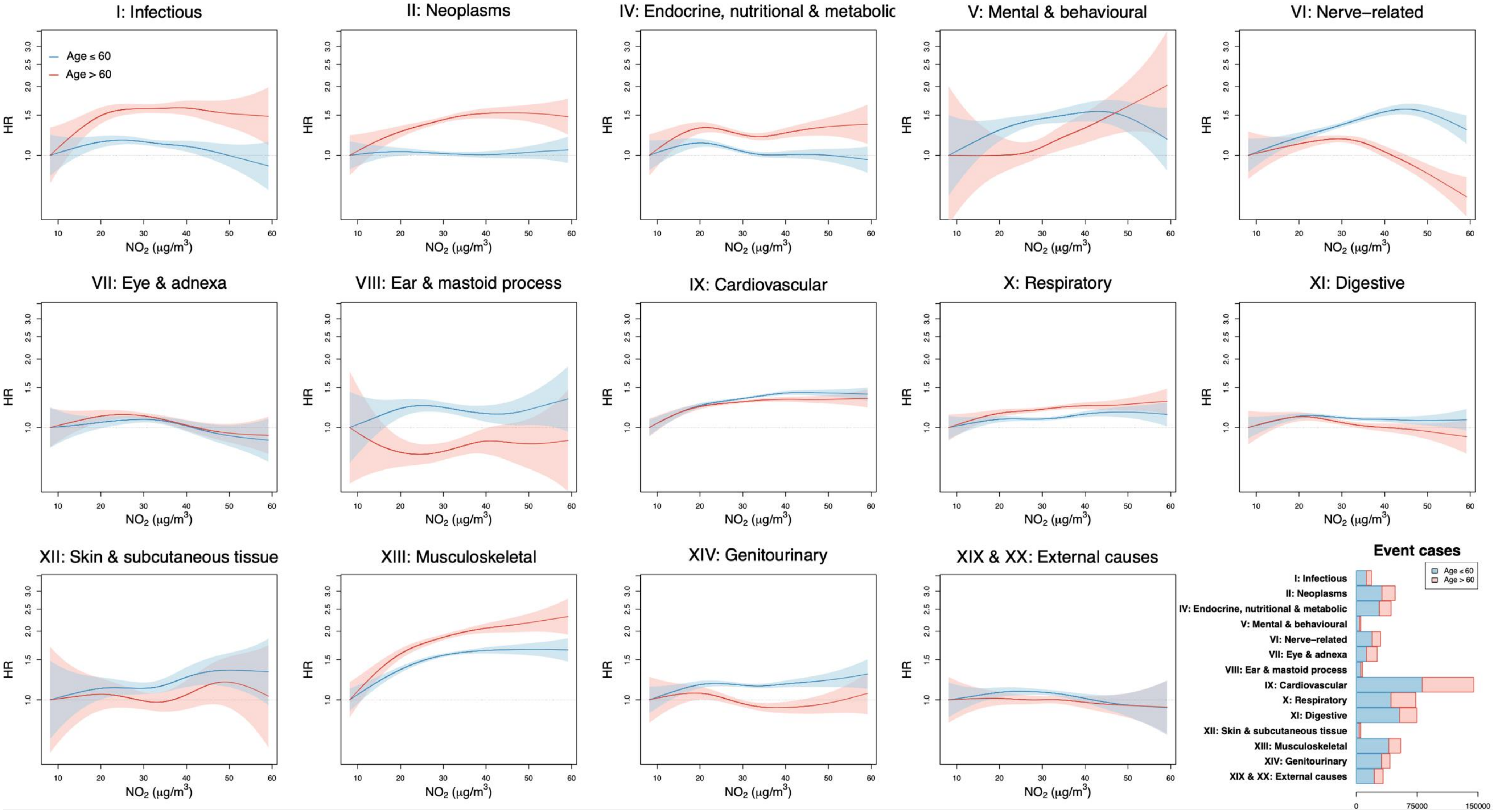

eFigure 6. Hazard ratios (HR) and 95% confidence intervals (CIs) for incidence of 14 ICD-10 chapter-based endpoints per 10 μg/m^3 higher annual ambient NO_2 exposure in the main models stratified by age

Note: The solid boxes represent HRs, with the size inversely proportional to the variance of the logarithm of the HR, and the horizontal lines represent 95% CIs. Cochrane's Q test was used to calculate P values for heterogeneity between subgroups. All models were stratified by age-at-risk (in 1-year scale), ten study areas, and sex, and were adjusted for education, household income, smoking status, alcohol drinking, cooking fuel type, heating fuel type, self-rated health status, body mass index (BMI), physical activity level, temperature and relative humidity. The models fit from the 1th to 99th percentiles of NO_2 concentration. Abbreviations: CI, confidence interval; HR, hazard ratio.

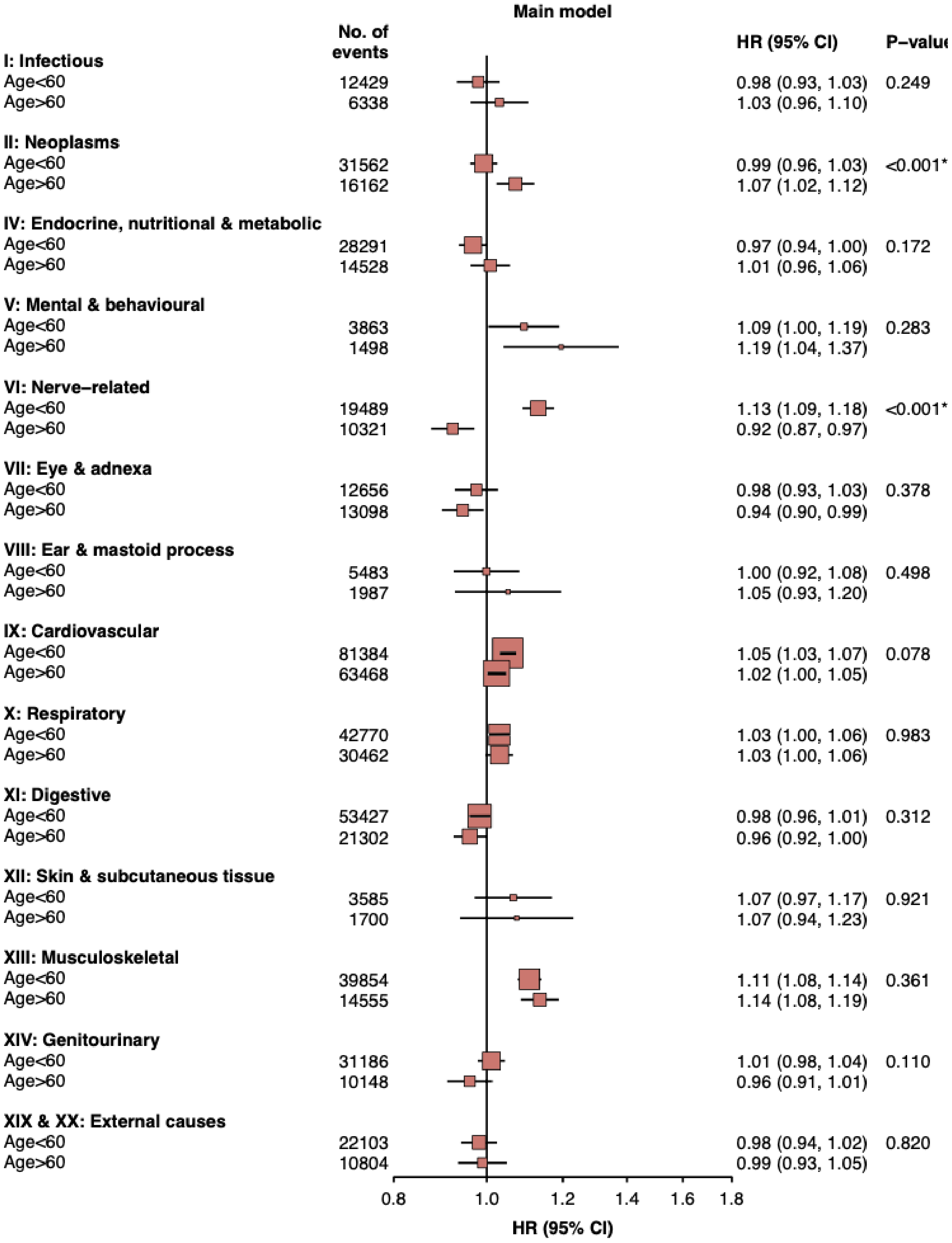

eFigure 7. Exposure-response relationships of annual ambient NO_2 exposure with incidence of 14 ICD-10 chapter-based endpoints in the main models stratified by sex

Note: Solid line represents HRs and the ribbon represents its 95% CI. All models were stratified by age-at-risk (in 1-year scale) and ten study areas, and were adjusted for education, household income, smoking status, alcohol drinking, cooking fuel type, heating fuel type, self-rated health status, body mass index (BMI), physical activity level, temperature and relative humidity. Abbreviations: CI, confidence interval; HR, hazard ratio.

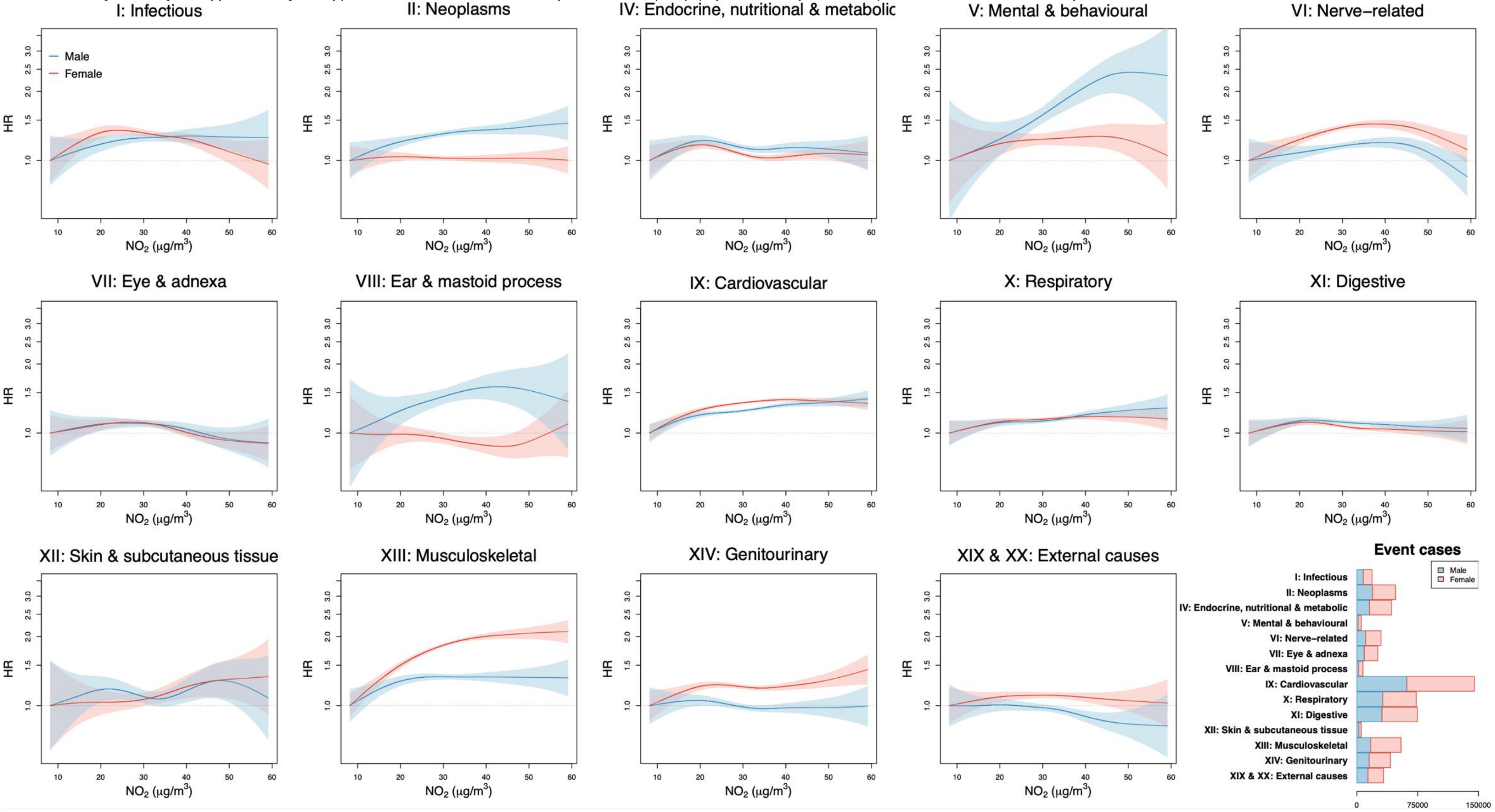

eFigure 8. Hazard ratios (HR) and 95% confidence intervals (CIs) for incidence of 14 ICD-10 chapter-based endpoints per 10 μg/m³ higher annual ambient NO_2 exposure in the main models stratified by sex

Note: The solid boxes represent HRs, with the size inversely proportional to the variance of the logarithm of the HR, and the horizontal lines represent 95% CIs. Cochrane's Q test was used to calculate P values for heterogeneity between subgroups. All models were stratified by age-at-risk (in 1-year scale) and ten study areas, and were adjusted for education, household income, smoking status, alcohol drinking, cooking fuel type, heating fuel type, self-rated health status, body mass index (BMI), physical activity level, temperature and relative humidity. The models fit from the 1th to 99th percentiles of NO_2 concentration. Abbreviations: CI, confidence interval; HR, hazard ratio.

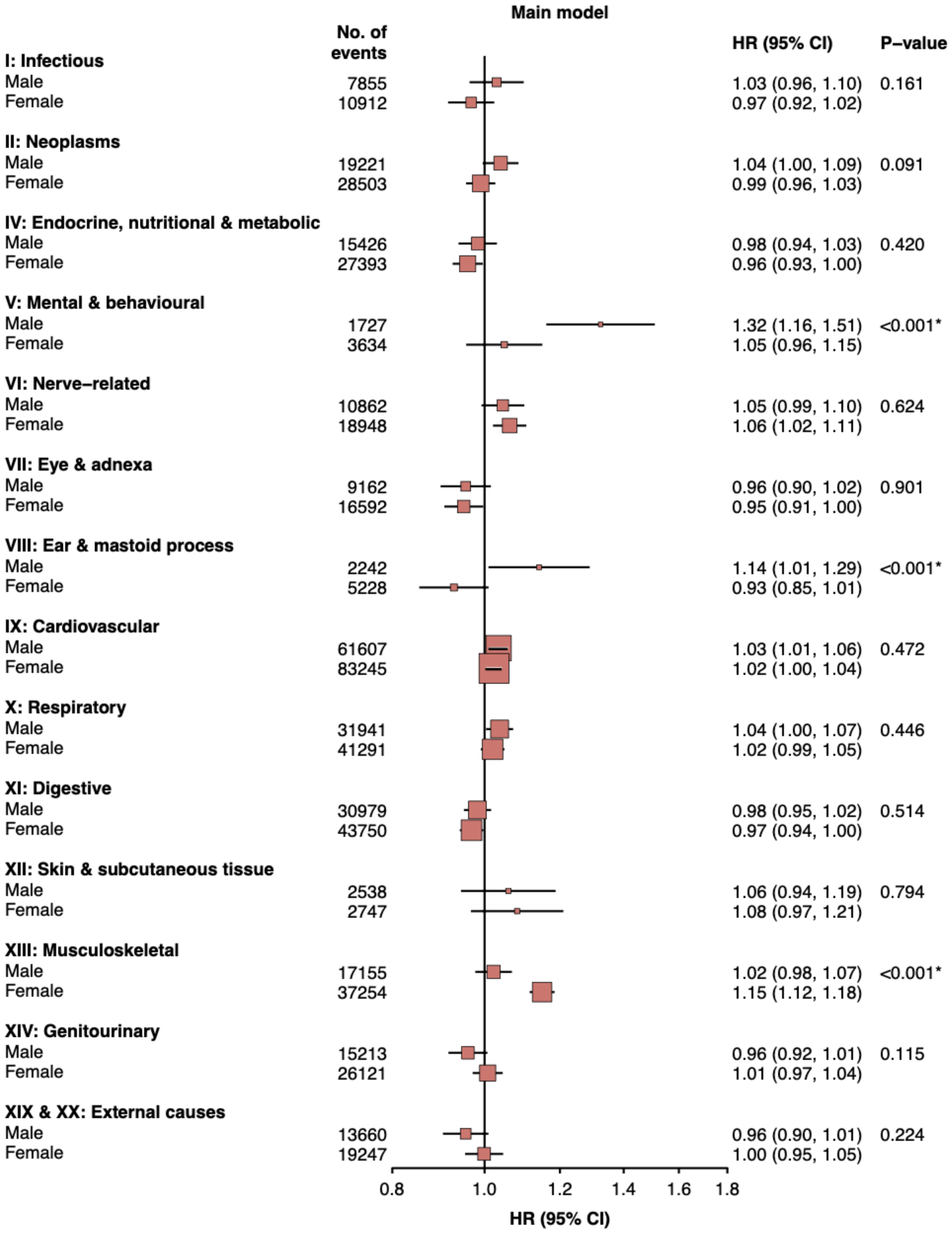

eFigure 9. Exposure-response relationships of annual ambient NO_2 exposure with incidence of 14 ICD-10 chapter-based endpoints in the main models stratified by smoking status

Note: Never-regular smokers: never-regular, occasional; Ever-regular smokers: ex-regular, current-regular. Solid line represents HRs and the ribbon represents its 95% CI. All models were stratified by age-at-risk (in 1-year scale), ten study areas, and sex, and were adjusted for education, household income, alcohol drinking, cooking fuel type, heating fuel type, self-rated health status, body mass index (BMI), physical activity level, temperature and relative humidity. Abbreviations: CI, confidence interval; HR, hazard ratio.

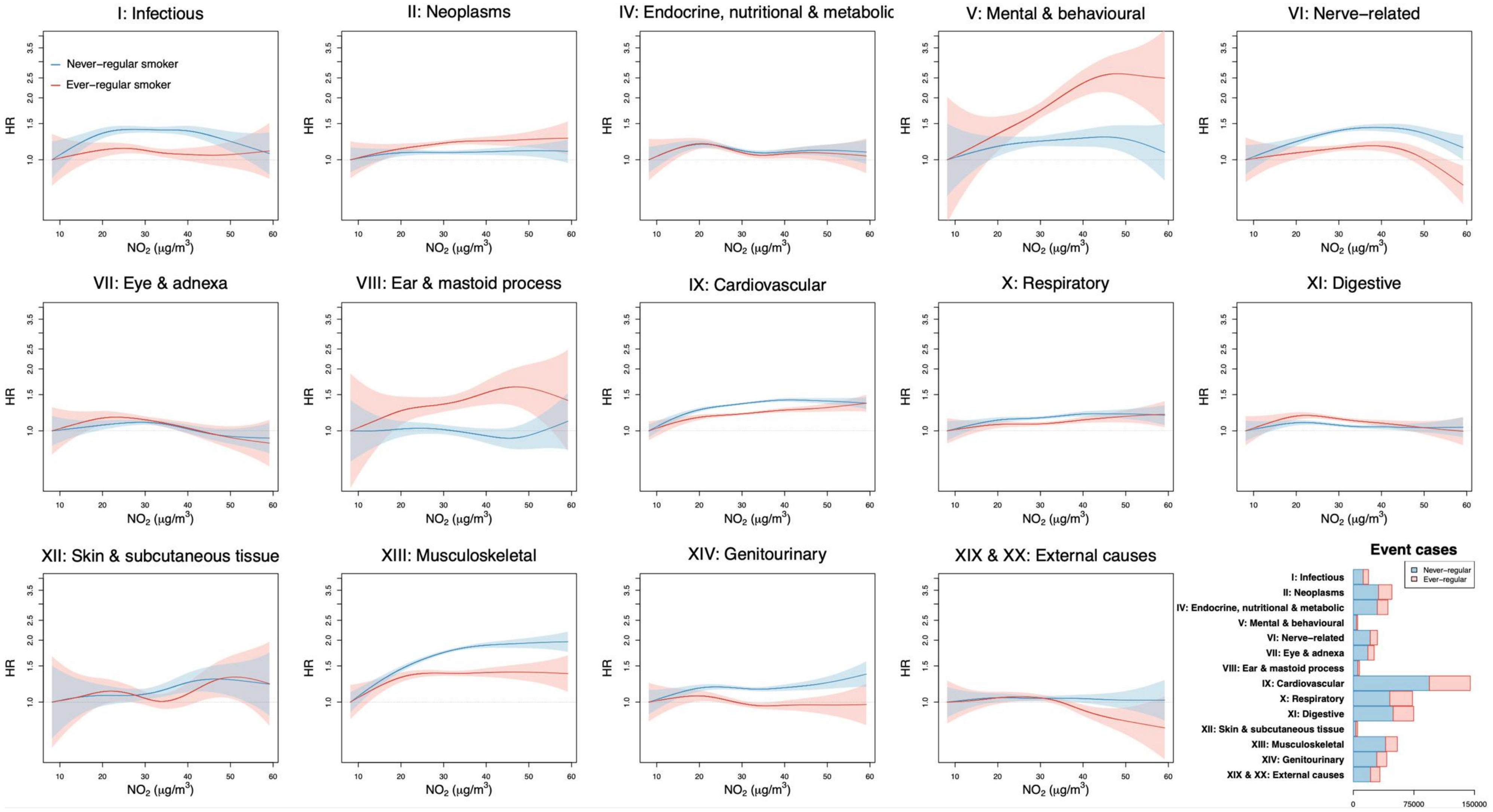

eFigure 10. Hazard ratios (HR) and 95% confidence intervals (CIs) for incidence of 14 ICD-10 chapter-based endpoints per 10 μg/m³ higher annual ambient NO_2 exposure in the main models stratified by smoking status

Note: Never-regular smokers: never-regular, occasional; Ever-regular smokers: ex-regular, current-regular. The solid boxes represent HRs, with the size inversely proportional to the variance of the logarithm of the HR, and the horizontal lines represent 95% CIs. Cochrane's Q test was used to calculate P values for heterogeneity between subgroups. All models were stratified by age-at-risk (in 1-year scale), ten study areas, and sex, and were adjusted for education, household income, alcohol drinking, cooking fuel type, heating fuel type, self-rated health status, body mass index (BMI), physical activity level, temperature and relative humidity. The models fit from the 1th to 99th percentiles of NO_2 concentration. Abbreviations: CI, confidence interval; HR, hazard ratio.

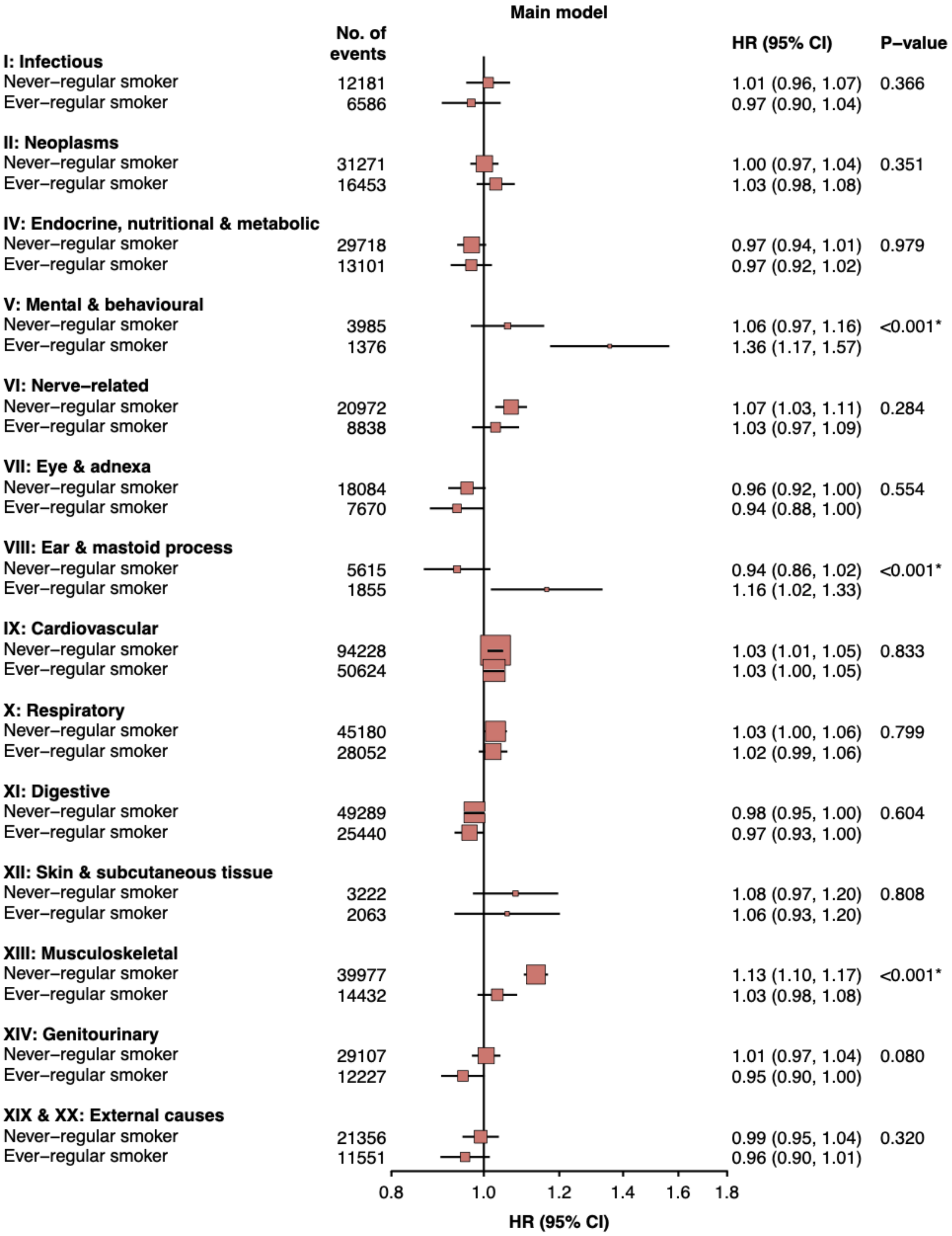

eFigure 11. Exposure-response relationships of annual ambient NO_2 exposure with incidence of 14 ICD-10 chapter-based endpoints in the main models stratified by physical activity levels

Note: Solid line represents HRs and the ribbon represents its 95% CI. All models were stratified by age-at-risk (in 1-year scale), ten study areas, and sex, and were adjusted for education, household income, smoking status, alcohol drinking, cooking fuel type, heating fuel type, self-rated health status, body mass index (BMI), temperature and relative humidity. Abbreviations: CI, confidence interval; HR, hazard ratio.

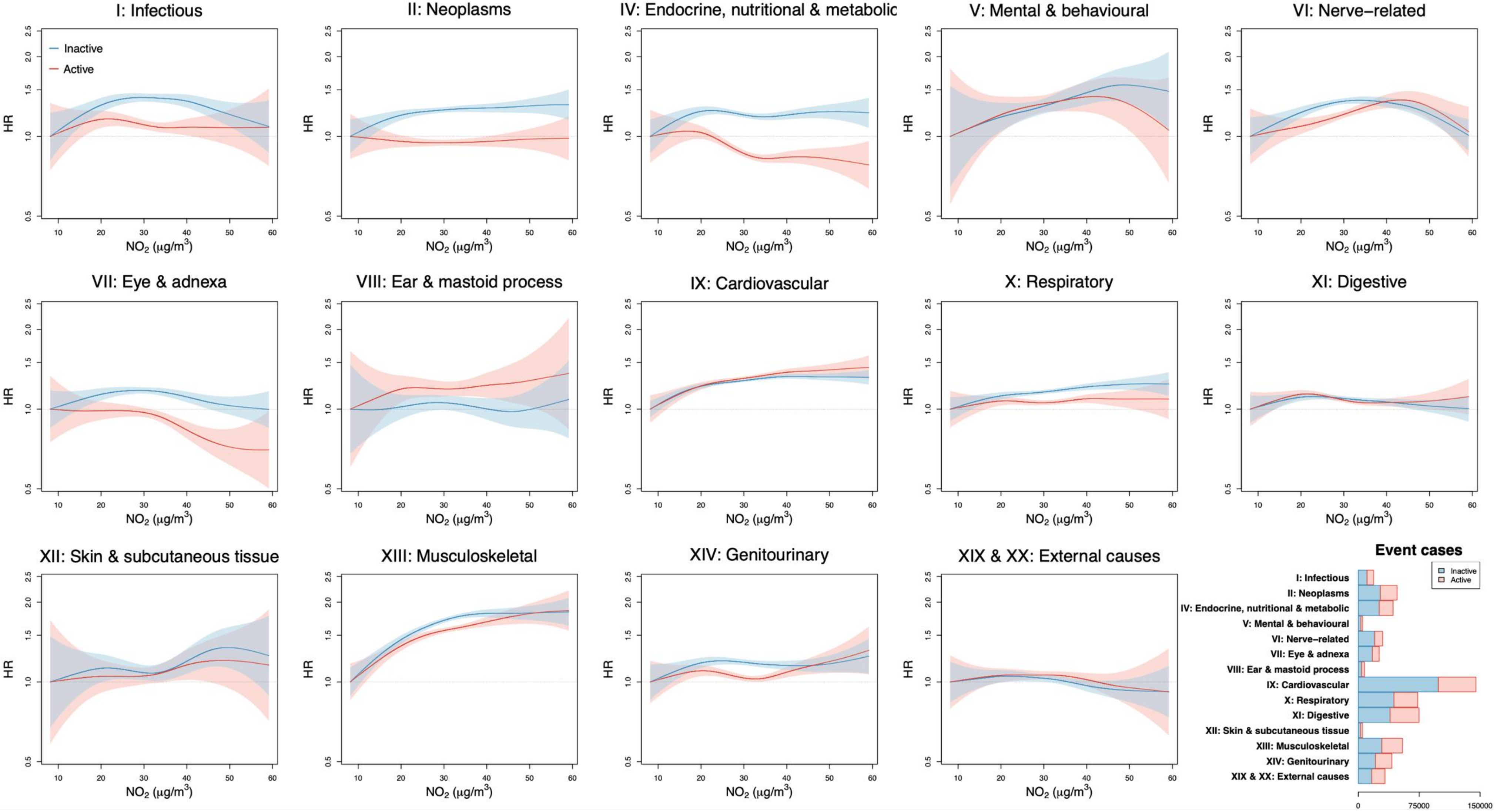

eFigure 12. Hazard ratios (HR) and 95% confidence intervals (CIs) for incidence of 14 ICD-10 chapter-based endpoints per 10 μg/m^3 higher annual ambient NO_2 exposure in the main models stratified by physical activity levels

Note: The solid boxes represent HRs, with the size inversely proportional to the variance of the logarithm of the HR, and the horizontal lines represent 95% CIs. Cochrane's Q test was used to calculate P values for heterogeneity between subgroups. All models were stratified by age-at-risk (in 1-year scale), ten study areas, and sex, and were adjusted for education, household income, smoking status, alcohol drinking, cooking fuel type, heating fuel type, self-rated health status, body mass index (BMI), temperature and relative humidity. The models fit from the 1th to 99th percentiles of NO_2 concentration. Abbreviations: CI, confidence interval; HR, hazard ratio.

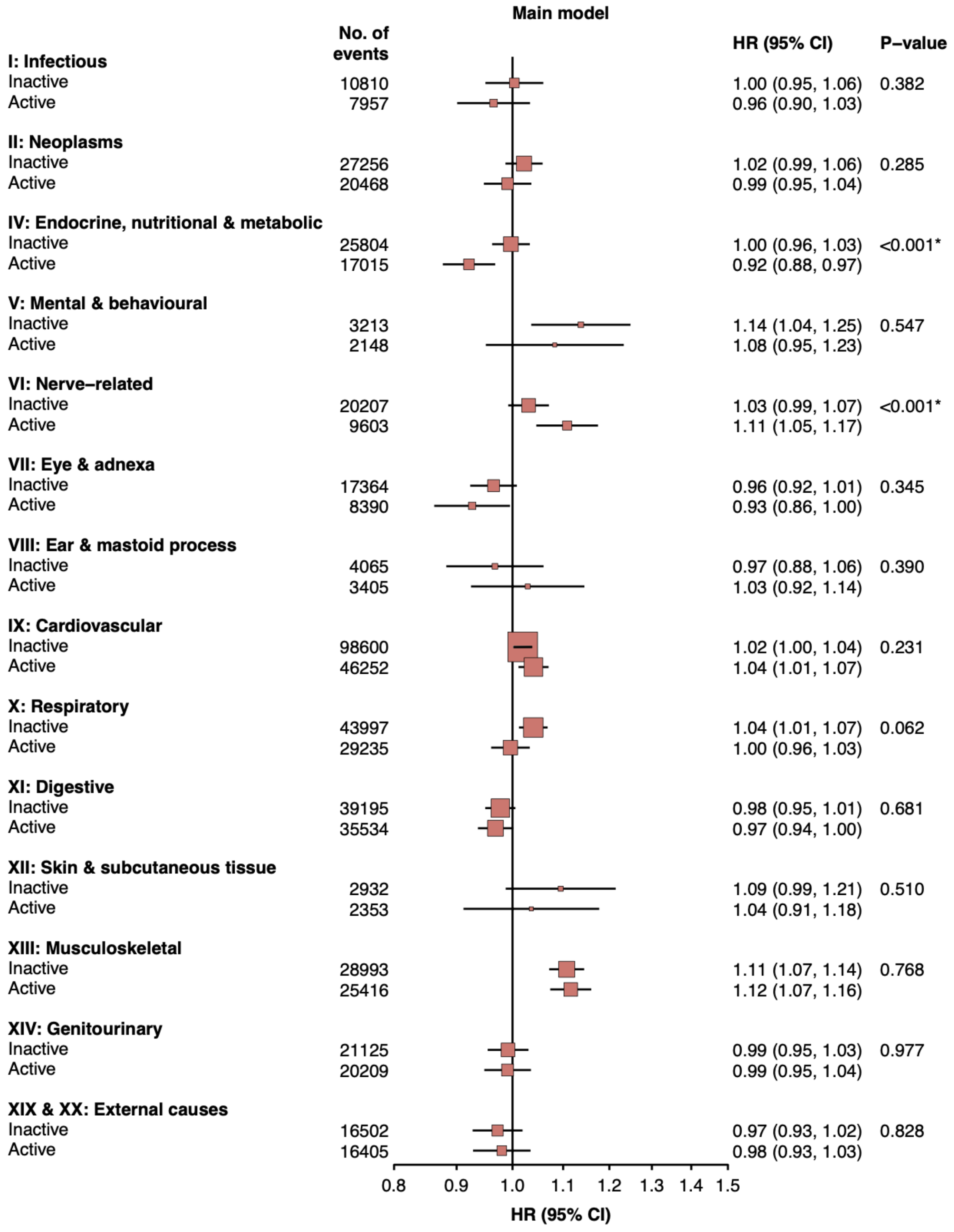

eFigure 13. Exposure-response relationships of annual ambient NO_2 exposure with incidence across 14 ICD-10 chapter-based endpoints in the main models stratified by drinking status

Note: Never-regular drinkers: Never-regular, occasional or seasonal, or monthly intake; Ever-regular drinkers: ex-regular, reduced intake, weekly. Solid line represents HRs and the ribbon represents its 95% CI. All models were stratified by age-at-risk (in 1-year scale), ten study areas, and sex, and were adjusted for education, household income, smoking status, cooking fuel type, heating fuel type, self-rated health status, body mass index (BMI), physical activity level, temperature and relative humidity. Abbreviations: CI, confidence interval; HR, hazard ratio.

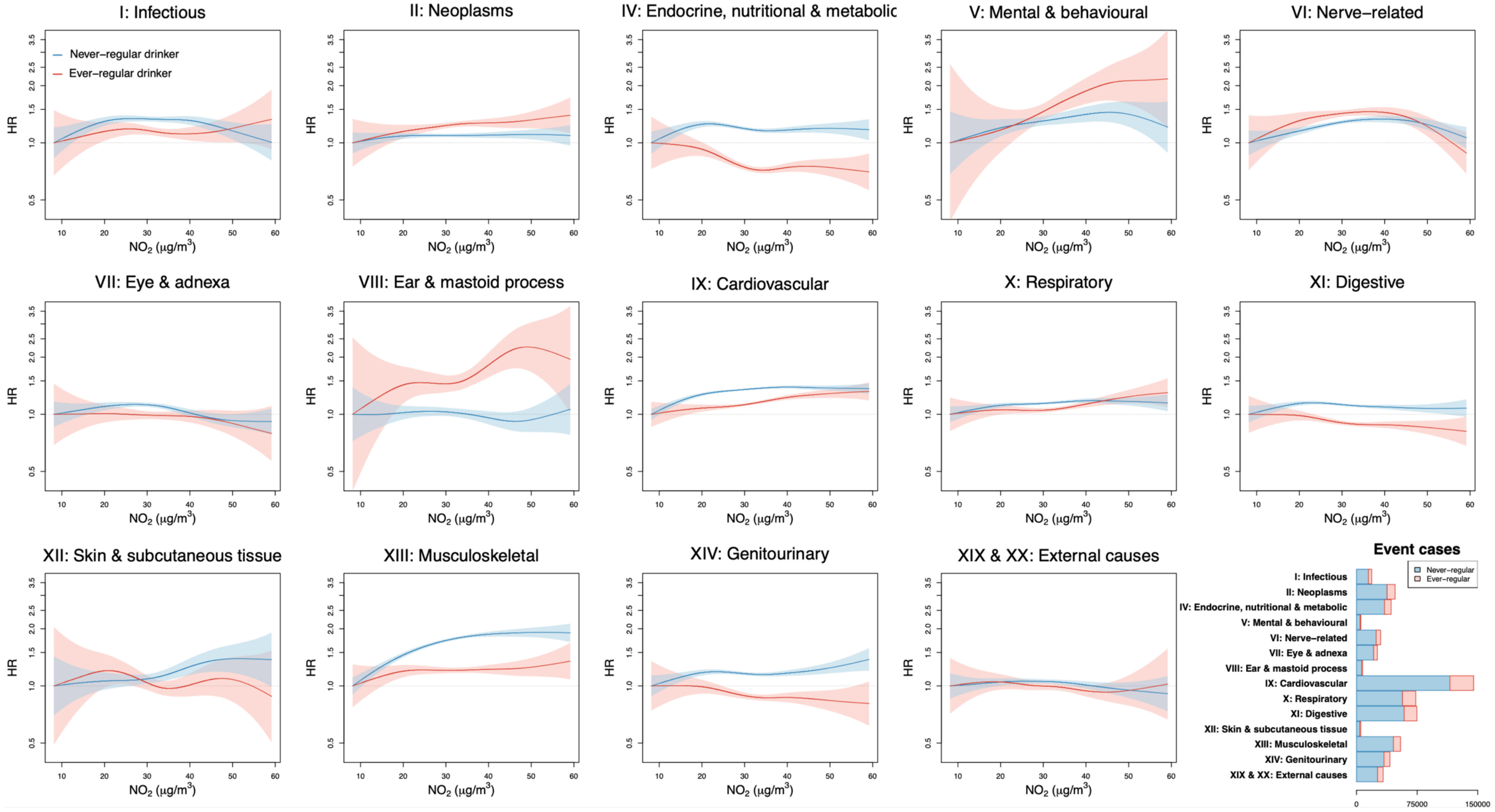

eFigure 14. Hazard ratios (HR) and 95% confidence intervals (CIs) for incidence of 14 ICD-10 chapter-based endpoints per 10 μg/m^3 higher annual ambient NO_2 exposure in the main models stratified by drinking status

Note: Never-regular drinkers: Never-regular, occasional or seasonal, or monthly intake; Ever-regular drinkers: ex-regular, reduced intake, weekly. The solid boxes represent HRs, with the size inversely proportional to the variance of the logarithm of the HR, and the horizontal lines represent 95% CIs. Cochrane's Q test was used to calculate P values for heterogeneity between subgroups. All models were stratified by age-at-risk (in 1-year scale), ten study areas, and sex, and were adjusted for education, household income, smoking status, cooking fuel type, heating fuel type, self-rated health status, body mass index (BMI), physical activity level, temperature and relative humidity. The models fit from the 1th to 99th percentiles of NO_2 concentration. Abbreviations: CI, confidence interval; HR, hazard ratio.

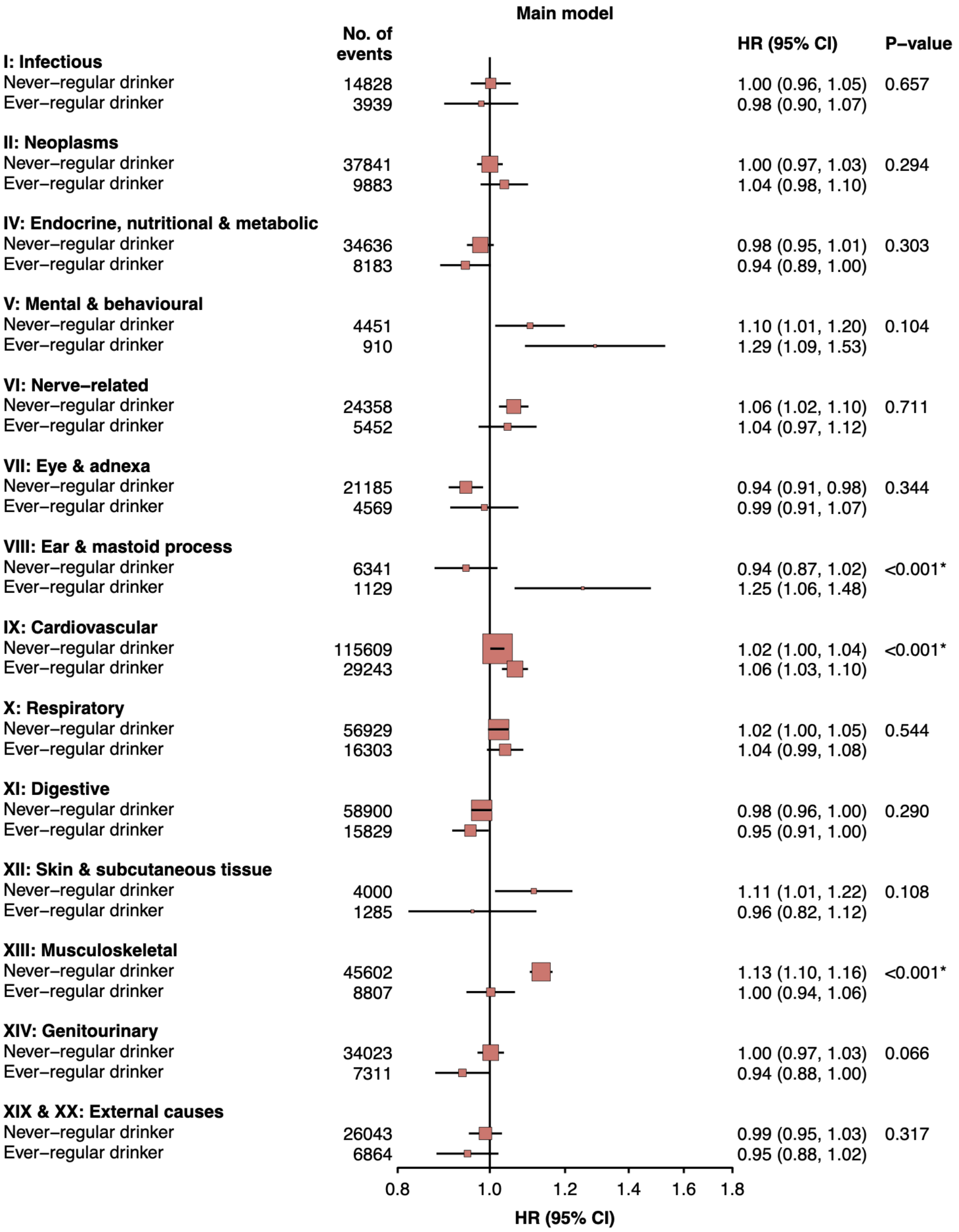

eFigure 15. Exposure-response relationships of annual ambient NO_2 exposure with incidence of musculoskeletal and mental & behavioural disease in the main models stratified by smoking and drinking status

Note: Never-regular smokers: never-regular, occasional; Ever-regular smokers: ex-regular, current-regular. Never-regular drinkers: Never-regular, occasional or seasonal, or monthly intake; Ever-regular drinkers: ex-regular, reduced intake, weekly. Solid line represents HRs and the ribbon represents its 95% CI. All models were stratified by age-at-risk (in 1-year scale), ten study areas, and sex, and were adjusted for education, household income, smoking status (if applicable), alcohol drinking (if applicable), cooking fuel type, heating fuel type, self-rated health status, body mass index (BMI), physical activity level, temperature and relative humidity. Abbreviations: CI, confidence interval; HR, hazard ratio.

XIII: Musculoskeletal disease

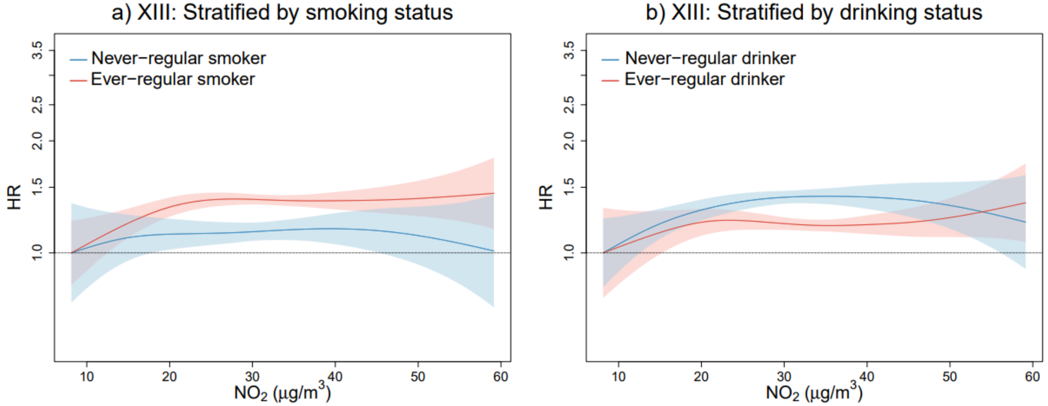

V: Mental & behavioural disease

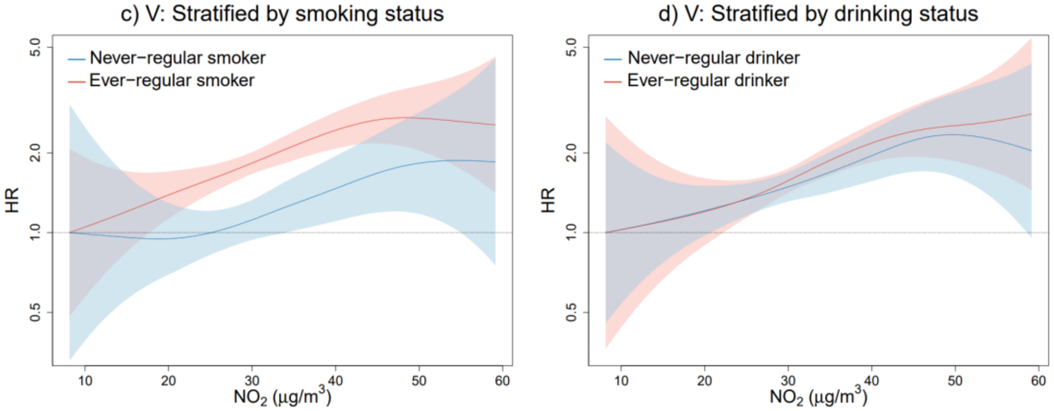

eFigure 16. Hazard ratios (HR) and 95% confidence intervals (CIs) for incidence of 14 ICD-10 chapter-based endpoints per 10 μg/m$_3$ higher annual ambient NO_2 exposure in the main models and main + $PM_{2.5}$ two-pollutant models after excluding participants with relevant disease incidence during the first three years of follow-up

Note: The solid boxes represent HRs, with the size inversely proportional to the variance of the logarithm of the HR, and the horizontal lines represent 95% Cis. All models were stratified by age-at-risk (in 1-year scale), ten study areas, and sex, and were adjusted for education, household income, smoking status, alcohol drinking, cooking fuel type, heating fuel type, self-rated health status, body mass index (BMI), physical activity level, temperature and relative humidity. The models fit from the 1th to 99th percentiles of NO_2 concentration. Abbreviations: CI, confidence interval; HR, hazard ratio.

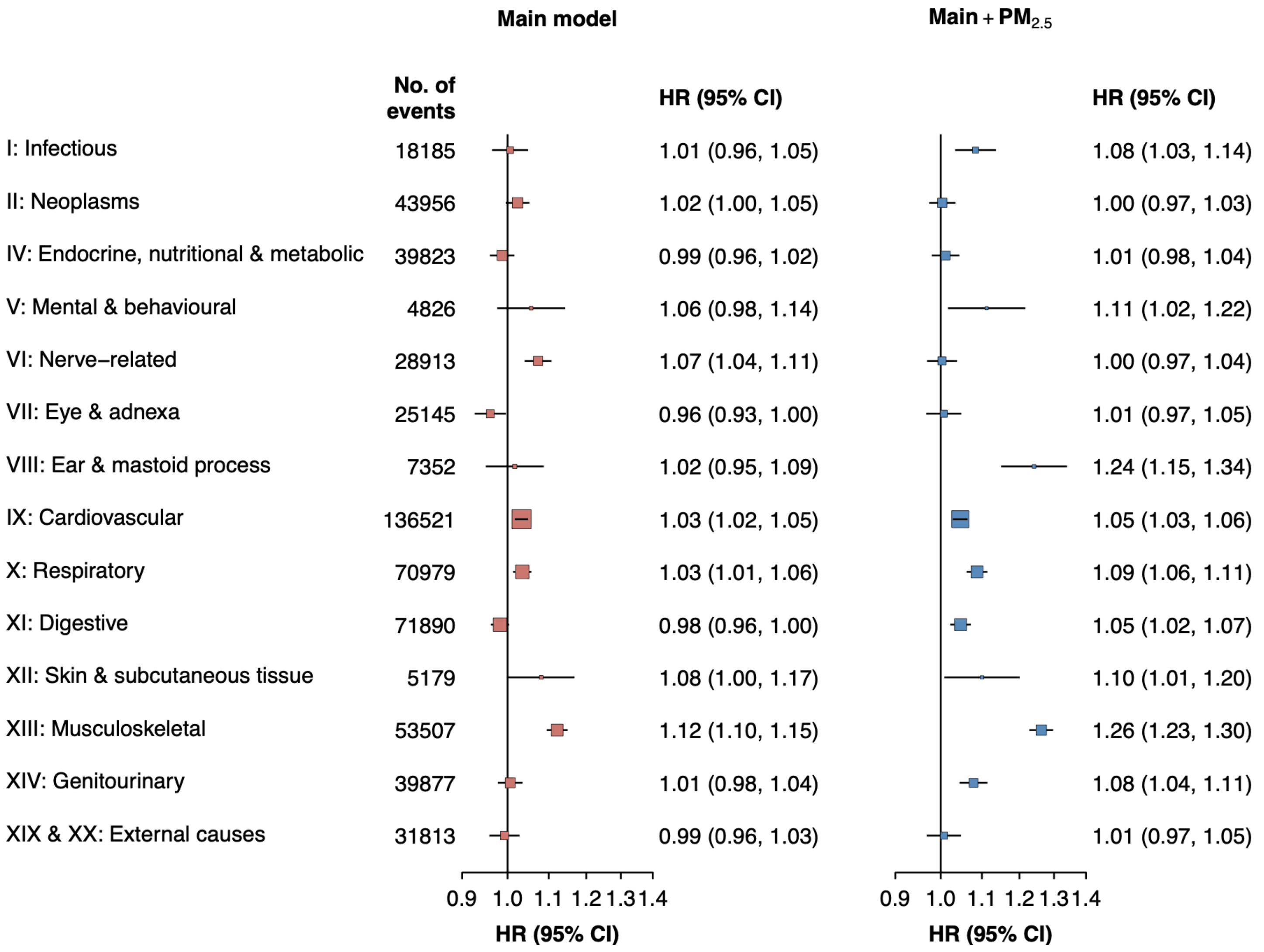

eFigure 17. Hazard ratios (HR) and 95% confidence intervals (CIs) for incidence of eight ICD-10 chapter-based endpoints per 10 μg/m^3 higher annual ambient NO_2 exposure in the main models and main + $PM_{2.5}$ two-pollutant models after excluding participants with relevant prior medical history at baseline

Note: The solid boxes represent HRs, with the size inversely proportional to the variance of the logarithm of the HR, and the horizontal lines represent 95% CIs. As the previous medical history recorded at baseline were only involved in eight chapters, the sensitivity analyses were only conducted for these chapters. The exclusions were: any prior cancer for neoplasms; diabetes for endocrine, nutritional & metabolic diseases; psychiatric disorders for mental & behavioural diseases; coronary heart disease, stroke, transient ischaemic attack, and rheumatic heart disease for cardiovascular disease; chronic obstructive pulmonary disease, emphysema, chronic bronchitis, tuberculosis, and asthma for respiratory disease; cirrhosis, hepatitis, and peptic ulcer for digestive disease; fracture and rheumatic arthritis for musculoskeletal disease; kidney disease for genitourinary disease. All models were stratified by age-at-risk (in 1-year scale), ten study areas, and sex, and were adjusted for education, household income, smoking status, alcohol drinking, cooking fuel type, heating fuel type, self-rated health status, body mass index (BMI), physical activity level, temperature and relative humidity. The models fit from the 1th to 99th percentiles of NO_2 concentration. Abbreviations: CI, confidence interval; HR, hazard ratio.

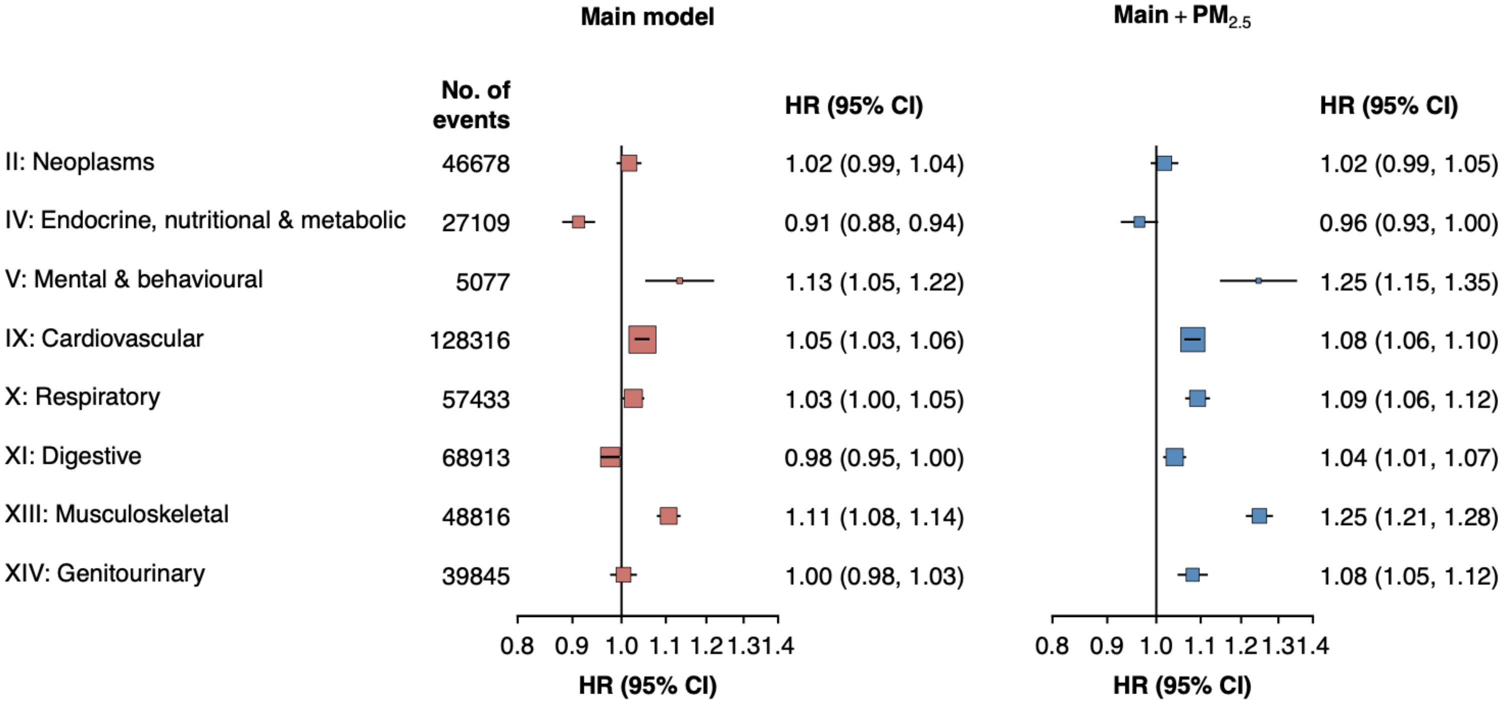

eFigure 18. Lag patterns for associations (hazard ratios) of long-term NO_2 exposure with incidence of 14 ICD-10 chapter-based endpoints in the main models

Note: The solid boxes represent HRs, with the size inversely proportional to the variance of the logarithm of the HR, and the horizontal lines represent 95% CIs. All models were stratified by age-at-risk (in 1-year scale), ten study areas, and sex, and were adjusted for education, household income, smoking status, alcohol drinking, cooking fuel type, heating fuel type, self-rated health status, body mass index (BMI), physical activity level, temperature and relative humidity. The models fit from the 1th to 99th percentiles of NO_2 concentration. Abbreviations: CI, confidence interval; HR, hazard ratio; Lag 0-3, three years lagged NO_2; Lag 0-5, five years lagged NO_2.

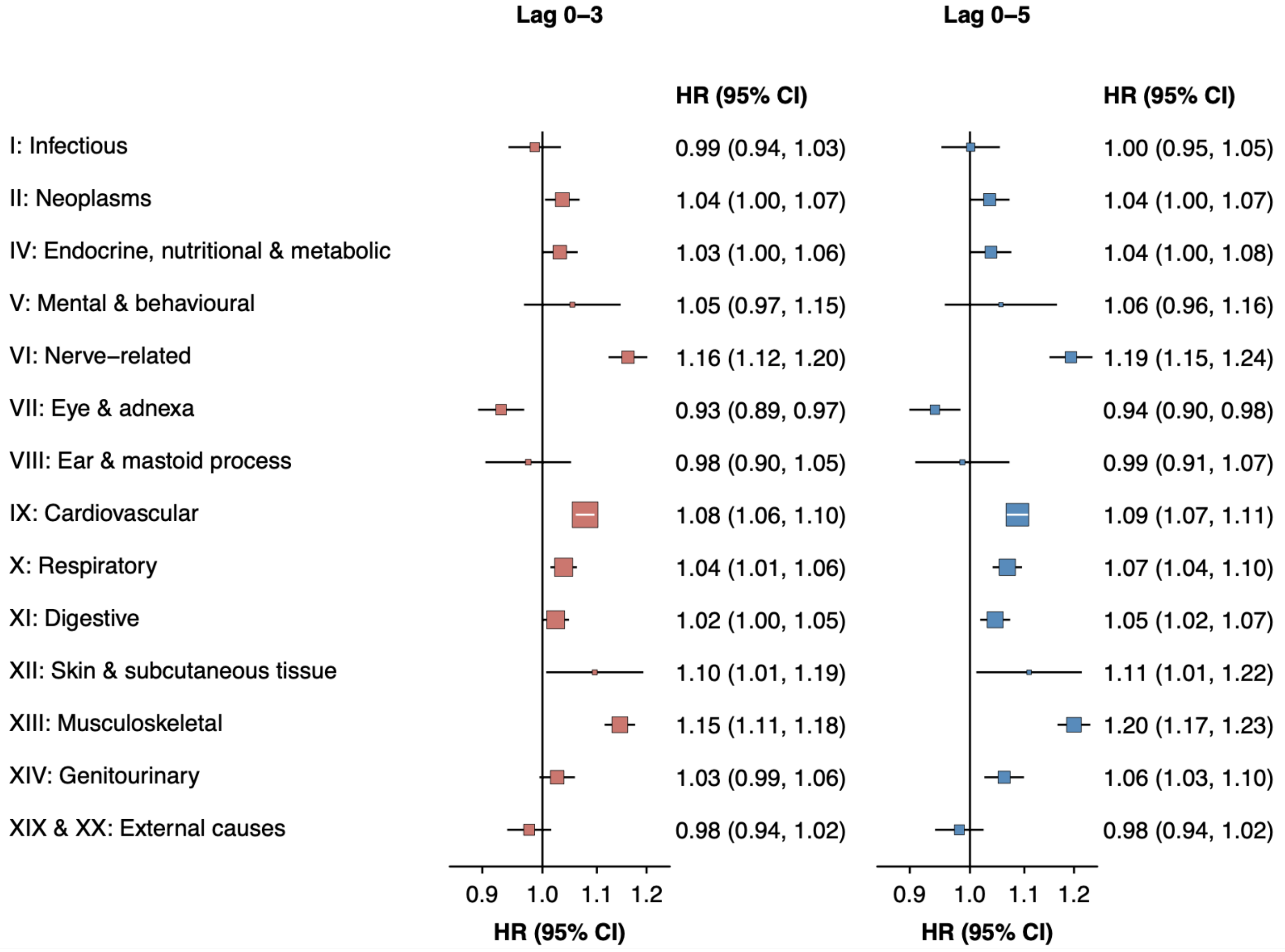

References for the appendix

1. Li X, Wang P, Wang W, et al. Mortality burden due to ambient nitrogen dioxide pollution in China: Application of high-resolution models. *Environ Int* 2023; **176**: 107967.
2. Yang L, Chen Z. Chapter 5: Monitoring long-term health outcomes of biobank participants by record linkages. In: Chen Z, ed. Population biobank studies: a practical guide. Singapore: Springer Nature Singapore Pte Ltd; 2020.
3. Chen Y, Clarke R. Verification and adjudication of health outcomes in prospective cohort studies. In: Chen Z, ed. Population biobank studies: a practical guide. Singapore: Springer Nature SingaporePte Ltd; 2020.
4. China Kadoorie Biobank. Disease phenotyping. 2022. https://www.ckbiobank.org/achievements/disease-phenotyping (accessed 5 October 2024.
5. Chen Z, Emberson J, Collins R. Strategic Need for Large Prospective Studies in Different Populations. *JAMA* 2020; **323**(4): 309-10.
6. Hong LS, Lewington S. Lexis Expansion- Age-at-risk adjustment for survival analysis. SAS Conference Proceedings: Pharmaceutical Users Software Exchange 2013; 2013; Brussels, Belgium; 2013.
7. Bennett DA, Du H, Clarke R, et al. Association of Physical Activity With Risk of Major Cardiovascular Diseases in Chinese Men and Women. *JAMA Cardiol* 2017; **2**(12): 1349-58.
8. Du H, Li L, Whitlock G, et al. Patterns and socio-demographic correlates of domain-specific physical activities and their associations with adiposity in the China Kadoorie Biobank study. *BMC Public Health* 2014; **14**: 826.
9. Ainsworth BE, Haskell WL, Herrmann SD, et al. 2011 Compendium of Physical Activities: a second update of codes and MET values. *Med Sci Sports Exerc* 2011; **43**(8): 1575-81.
10. Gowers AM, Walton H, Exley KS, Hurley JF. Using epidemiology to estimate the impact and burden of exposure to air pollutants. *Philos Trans A Math Phys Eng Sci* 2020; **378**(2183): 20190321.
11. COMEAP. Associations of long-term average concentrations of nitrogen dioxide with mortality. London, UK: Public Health England, 2018.
